# Optimization of AAV vectors for transactivator-regulated enhanced gene expression within targeted neuronal populations

**DOI:** 10.1016/j.isci.2024.109878

**Published:** 2024-05-03

**Authors:** Leo Kojima, Kaoru Seiriki, Hiroki Rokujo, Takanobu Nakazawa, Atsushi Kasai, Hitoshi Hashimoto

**Affiliations:** 1 Laboratory of Molecular Neuropharmacology, Graduate School of Pharmaceutical Sciences, Osaka University, Suita, Osaka 565-0871, Japan; 2 Department of Bioscience, Tokyo University of Agriculture, Setagaya-ku, Tokyo 156-8502, Japan; 3 Systems Neuropharmacology, Research Institute of Environmental Medicine, Nagoya University, Nagoya 464-8601, Japan; 4 Institute for Open and Transdisciplinary Research Initiatives, Osaka University, Suita, Osaka 565-0871, Japan; 5 Molecular Research Center for Children’s Mental Development, United Graduate School of Child Development, Osaka University, Kanazawa University, Hamamatsu University School of Medicine, Chiba University and University of Fukui, Suita, Osaka 565-0871, Japan; 6 Institute for Datability Science, Osaka University, Suita, Osaka 565-0871, Japan; 7 Department of Molecular Pharmaceutical Sciences, Graduate School of Medicine, Osaka University, Suita, Osaka 565-0871, Japan

**Keywords:** Molecular biology, Neuroscience

## Abstract

Adeno-associated virus (AAV) vectors are potential tools for cell-type-selective gene delivery to the central nervous system. Although cell-type-specific enhancers and promoters have been identified for AAV systems, there is limited information regarding the effects of AAV genomic components on the selectivity and efficiency of gene expression. Here, we offer an alternative strategy to provide specific and efficient gene delivery to a targeted neuronal population by optimizing recombinant AAV genomic components, named TAREGET (TransActivator-Regulated Enhanced Gene Expression within Targeted neuronal populations). We established this strategy in oxytocinergic neurons and showed that the TAREGET enabled sufficient gene expression to label long-projecting axons in wild-type mice. Its application to other cell types, including serotonergic and dopaminergic neurons, was also demonstrated. These results demonstrate that optimization of AAV expression cassettes can improve the specificity and efficiency of cell-type-specific gene expression and that TAREGET can renew previously established cell-type-specific promoters with improved performance.

## Highlights

• AAV expression cassettes were optimized for cell-type specificity and efficiency
• The Tet-Off system is more suitable for the optimized AAV than Cre recombinase
• The optimized AAV system was applied to brain-wide axonal labeling and genome editing

## Graphical abstract

**Figure:**
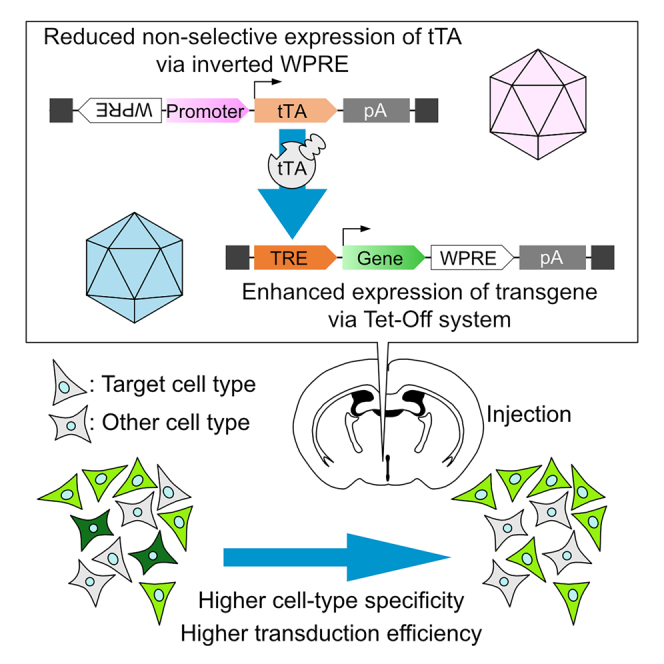

## Introduction

The brain is composed of heterogeneous neuronal populations, including glutamatergic, GABAergic, monoaminergic, and neuropeptidergic neurons. Classification and functional analysis of each cell type are prerequisites for a precise understanding of brain function and pathologies in brain diseases.^1^^,^^2^ To achieve this, transgenic or knock-in animals have been engineered to express exogenous genes such as fluorescent proteins and Cre recombinase in specific neuronal populations to label or manipulate the target cell type.^3^^,^^4^ Despite the high utility of cell-type-selective transgenic animals, several limitations of these tools have been reported, including the huge effort required to breed transgenic animals, unintentional off-target and insertional effects,^5^^,^^6^^,^^7^^,^^8^^,^^9^^,^^10^ and insufficient efficiency for labeling.^11^ Backcrossing to the desired genetic background is also a time-consuming task when the target animal strains differ from the available genetic backgrounds.

Recently, various adeno-associated virus (AAV) vectors that utilize cell-type-specific promoters^12^^,^^13^^,^^14^^,^^15^^,^^16^ or enhancers^17^^,^^18^^,^^19^^,^^20^ have been developed in order to deliver genes into the targeted cell types and investigate their anatomical and functional characteristics as alternative strategies for transgenic animals. However, in some cases, the selectivity and efficiency of targeted gene expression in the AAV-based approach have been reported to be relatively lower than those in Cre lines.^10^^,^^19^^,^^21^ Moreover, although some cell-type-specific promoters for neuromodulator-expressing neurons have been identified, most have a gene length of 2–3 kilobases (kb),^14^^,^^16^^,^^21^^,^^22^ which occupies approximately half of the assumed capable genome length of AAV (4.7 kb).^23^^,^^24^ These limitations may prevent the application of the AAV-based cell-type-specific approaches to broader experimental designs.

Oxytocin (OXT) neurons are involved in a wide range of physiological phenomena including social and nurturing behaviors.^25^^,^^26^^,^^27^^,^^28^ Reduced sociability can be associated with impaired OXT systems in some animal models, as we and others have previously identified reduced OXT or its receptor in mouse models of neurodevelopmental disorders that harbor genetic mutations or deletions of genes associated with human autism spectrum disorder.^29^^,^^30^^,^^31^ Thus, viral-genetic targeting of OXT neurons in genetic mouse models or inbred mouse strains for diseases may help understand the anatomical and functional properties of OXT neurons in healthy and diseased states. AAV-based cell-type-specific approaches for OXT neurons have been well established^32^^,^^33^^,^^34^ and reproduced in multiple independent studies.^28^^,^^35^ These applications mainly employ a single AAV vector system in which the OXT promoter directly drives the expression of transgenes. Although dual AAV vector systems carrying Cre/*loxP* or Tet-Off systems allow us to use a larger gene size, the selectivity for OXT neurons is reduced compared to the single AAV vector system.^36^ Alternative AAV strategies with improved versatility may shed light on the neuronal mechanisms underlying social behavior and impairment in animal models.

Here, we propose the transactivator-regulated enhanced gene expression within targeted neuronal populations (TAREGET) strategy for AAV-mediated cell-type-specific gene expression in wild-type mice. Using previously reported OXT promoters,^29^ we optimized the regulatory components of gene expression, thereby improving both the specificity and efficiency of gene expression via the Tet-Off system in OXT neurons. AAV TAREGET-OXT (TAREGET for OXT neurons) restricts the high expression levels of reporter fluorescent proteins within OXT neurons, and that allows whole-brain visualization of long-projecting OXT neuronal axons across multiple brain regions in wild-type mice. Furthermore, the TAREGET strategy is applicable to other cell types, such as serotonergic and dopaminergic neurons, by using their specific promoters. These results provide important insights into viral vector strategies targeting specific neuronal populations.

## Results

### Optimization of regulatory components in the AAV vector enables selective and robust gene expression in OXT neurons

**Figure 1:**
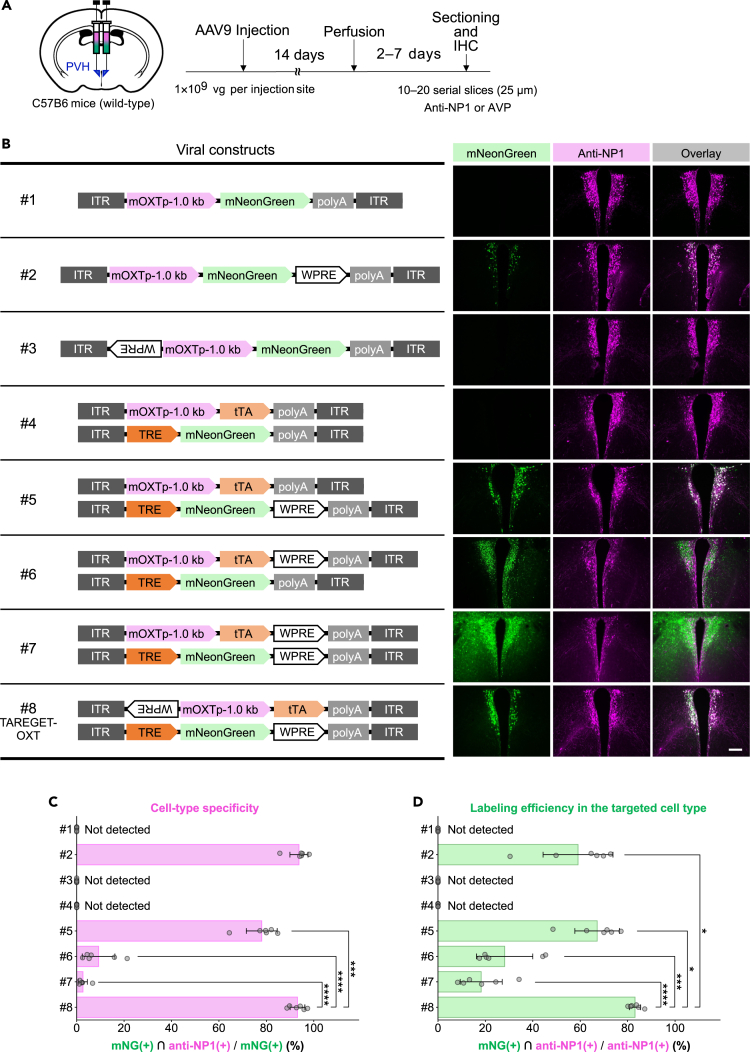
Optimization of the expression control system and regulatory component in recombinant AAV genomes using OXT promoter (A) Scheme of the experimental paradigm. C57BL/6N mice received stereotaxic injection of AAVs into the bilateral PVH regions. (B) AAV vector constructs and representative images of the PVH transduced by each AAV set. All images were obtained and presented with the same imaging and contrast conditions except for the mNG-channel in AAV set #7, because AAV set #7 induced high levels of mNG expression, and thereby single cells could not be identified due to signal saturation. Scale bar, 200 μm. (C) Quantitative analysis of specificity to the OXT neurons. Percentage of the number of mNG and NP1 (OXT neuronal marker) double-positive cells to that of mNG-positive cells was quantified. (D) Quantitative analysis of efficiency of gene expression in the OXT neurons. Percentage of the number of mNG and NP1 double-positive cells to that of NP1-positive cells was quantified. For C and D, the number on the vertical axis is corresponding to the number of AAV sets shown in B. PVH, paraventricular hypothalamic nucleus; IHC, immunohistochemistry; NP1, Neurophysin 1; AVP, arginine vasopressin; ITR, inverted terminal repeat; mOXTp mouse oxytocin promoter; mNG, mNeonGreen; WPRE, woodchuck hepatitis virus post-transcriptional regulatory element; polyA, polyadenylation signal; tTA, tetracycline transactivator; TRE, tetracycline response element. Statistical analysis was performed excluding groups in which mNG-positive cells were not detected. Point plots in C and D represent the quantified values per brain hemisphere (typically 6 regions from 3 mice). ∗*p* < 0.05; ∗∗*p* < 0.01; ∗∗∗*p* < 0.001; ∗∗∗∗*p* < 0.0001; n.s., not significant. Statistical significance is shown only for the comparisons between AAV set #8 (TAREGET-OXT) and other groups to avoid redundancy. Full statistics are shown in Table S1. Data are represented as mean ± SEM. See also Figure S1.

Previous studies suggest that inverted terminal repeat (ITR) sequences in the AAV genome can act as a promoter,^37^ and its undesired action may be attenuated by insertion of the woodchuck hepatitis virus post-transcriptional regulatory element (WPRE) with inverted orientation into the 5′ upstream of cell-type-selective promoter.^38^ Although the insertion of inverted WPRE is known to decrease gene expression at the mRNA level,^39^ these effects in AAV vectors have been less investigated quantitatively. To examine whether the position of regulatory components affects cell-type selectivity and compatibility to Tet-Off-mediated enhanced gene expression in OXT neurons, we compared eight patterns of AAV vector sets, all of which have a 1.0 kb fragment of the mouse OXT promoter (mOXTp)^29^ and reporter fluorescent protein, mNeonGreen (mNG),^40^ which is one of the brightest monomeric green fluorescent proteins in neuronal experiments. All AAV vectors were packaged into the AAV serotype 9 capsid and administered to the paraventricular nucleus of the hypothalamus (PVH) at a dose of 1 × 10^9^ vg via bilateral stereotaxic injection (Figure 1A). In the single AAV vector system (Figure 1B, AAV #1–3), insertion of WPRE into 3′ untranslated region (UTR) increased the number of mNG-positive cells in OXT neurons immunolabeled with antibody for Neurophysin 1 (NP1), which is a cleavage product from an OXT-precursor protein and a reliable marker for OXT neurons.^41^^,^^42^ AAV #2 carrying the 3′ WPRE achieved over 90% specificity of mNG expression in the OXT neurons (Figure 1C); however, only 59.1% of the NP1-positive cells were labeled with mNG (Figure 1D). These results suggest that in the single AAV system, the transcriptional activity of the 1.0 kb mOXTp is insufficient for labeling a large population of OXT neurons.

The low labeling efficiency can be attributed to the undetectable levels of mNG expression in the targeted neuronal populations. Given that the Tet system can be used not only for inducible expression of the target gene but also for the transcriptional amplification of cell-type-specific promoters,^43^^,^^44^ we examined whether using the Tet system would improve the expression levels of mNG controlled by the 1.0 kb mOXTp. For this purpose, we designed dual AAV vectors, which comprised a pair of AAVs carrying the tetracycline transactivator (tTA) under the mOXTp and mNG under the tetracycline response element (TRE) promoter, respectively (Figure 1B, AAV sets #4–8). The effects of WPRE in 3′ UTR with forward orientation and that in 5′ upstream of the promoter with inverted orientation were also evaluated in AAV sets #5–8. The combination of inverted WPRE at 5′ upstream of mOXTp and tTA (AAV set #8) showed the comparable selectivity for OXT neurons with the single AAV system #2 (93.1% vs. 93.8%; Figure 1C). Furthermore, the selectivity and labeling efficiency of AAV set #8 were higher than those of AAV set #5, which lacks the inverted WPRE at 5′ upstream of the promoter (Figure 1D), suggesting that the inverted WPRE can improve both cell-type specificity and efficiency of gene expression. Insertion of forward WPRE into the 3′ UTR of AAV sets #6 and 7, which enhances tTA expression, resulted in a greater overall number of mNG-expressing cells including non-OXT neurons (Figures S1A and S1B). Consequently, the specificity of mNG induction in OXT neurons was reduced in the AAV sets #6 and 7 compared to the AAV set #8 (Figure 1C). Despite the broader mNG expression, labeling efficiency in OXT neurons also decreased (Figure 1D). Given that extremely high-level expression of fluorescent proteins can be toxic,^45^ we evaluated the number of NP1-positive cells and cell death induction for AAV sets #6 and 7. These groups exhibited a reduction in the number of NP1-positive cells (Figures S1A–S1C) and an increase in the number of active caspase-3-positive cells (Figures S2A–S2C), suggesting that the reduction of mNG- and NP1-double-positive cells for AAV sets #6 and 7 was due to cytotoxicity. These results indicate that the dual AAV system, in which a tTA driver AAV carrying 5′ inverted WPRE and a TRE reporter AAV carrying gene of interests with WPRE in the 3′ UTR, can improve the selectivity and labeling efficiency of weak promoters for cell-type-specific gene expression. Therefore, we named this dual AAV system AAV TAREGET-OXT.

### AAV TAREGET yields cell-type-selective gene expression but requires optimal promoter activity for cell-type selectivity

**Figure 2:**
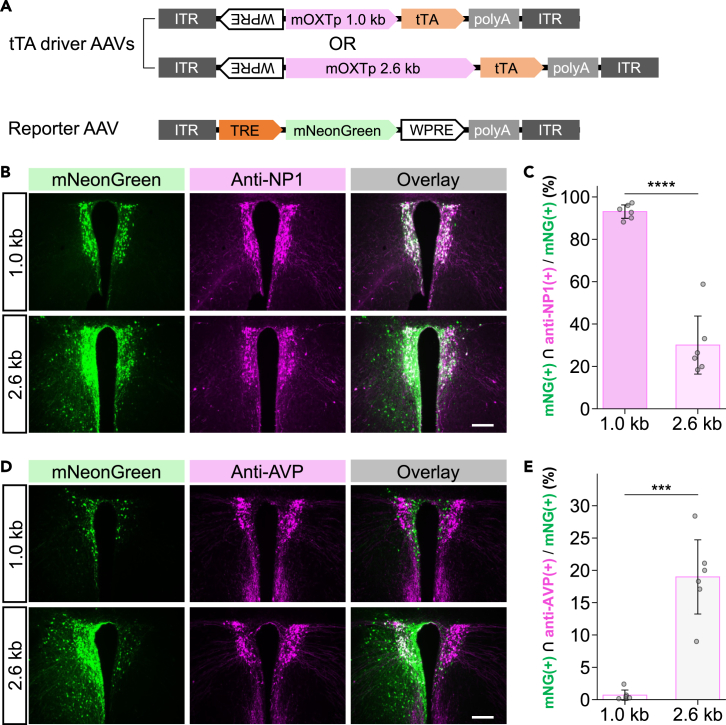
AAV TAREGET allows specific gene expression in OXT neurons but requires optimization of promoter activity (A) Scheme of AAV vector constructs to compare two types of mOXTp (1.0 kb and 2.6 kb) in the AAV TAREGET system. (B) Representative images of the PVH immunostained for NP1 (OXT neuron). (C) Quantitative comparison of specificity to the OXT neurons. The value of the 1.0 kb OXT promoter in Figure 1 was indicated for comparison. (D) Representative images of the PVH immunostained for AVP. (E) Quantitative comparison of leaky expression of mNG in the AVP neurons. Scale bars, 200 μm. ∗∗∗*p* < 0.001; ∗∗∗∗*p* < 0.0001. Data are represented as mean ± SEM. See also Figures S2 and S3.

To evaluate the robustness and scalability of the TAREGET strategy, we examined whether a different type of mOXT promoter in the AAV TAREGET system retains cell-type selectivity. Recent studies have shown that AAV carrying the 2–2.6 kb mOXT promoter drives selective gene expression in OXT neurons,^28^^,^^32^^,^^34^ and a variety of genes have been successfully transduced to OXT neurons selectively, including not only fluorescent proteins but also designer receptors exclusively activated by designer drugs (DREADD), hM3Dq,^28^ and a genetically encoded calcium indicator, GCaMP.^36^^,^^46^ Therefore, the longer mOXTp was considered to have more effective transcriptional activity than the 1.0 kb mOXTp. However, the use of strong promoters carries the risk of increased background expression when combined with the Tet-Off amplification system. We thus compared the previously used 1.0 kb OXT promoter with the stronger 2.6 kb promoter to determine whether the TAREGET strategy was applicable to stronger promoters for cell-type-specific labeling (Figure 2A). Although the dual AAV system carrying the 2.6 kb mOXTp yielded brighter fluorescent signals and labeled the majority of NP1-positive neurons in the PVH (Figures 2B and S3A), cell-type selectivity was decreased to approximately 30% (Figure 2C). Non-selective mNG expression in arginine vasopressin (AVP)-expressing neurons was also observed in 19.0% of mNG-positive neurons using the 2.6 kb mOXTp system, whereas less than 1.0% of mNG-positive neurons were AVP-positive in the 1.0 kb mOXTp system (Figures 2D, 2E, and S3B). The selectivity of the 1.0 kb mOXTp-based TAREGET was maintained throughout the anteroposterior axis (Figures S4A–S4C). These results suggest that the TAREGET strategy requires a promoter with optimal transcriptional activity for cell-type specificity.

### The principle of AAV-TAREGET for cell-type-selective experiments is applicable to Cre-lox system under the sparser labeling condition

**Figure 3:**
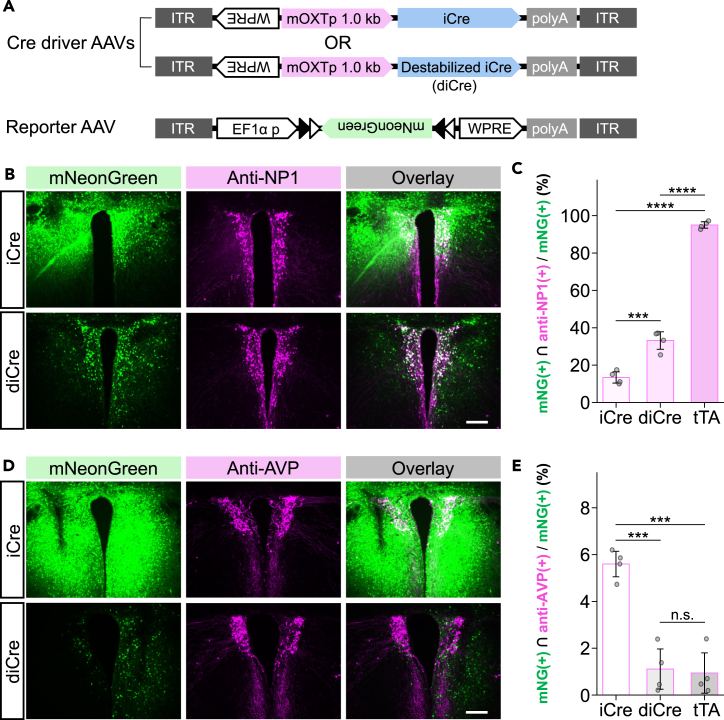
TAREGET strategy has greater suitability for the Tet-Off system compared to the Cre-lox expression system in OXT neurons (A) Scheme of AAV vector constructs utilizing iCre or destabilized iCre (diCre) in the AAV TAREGET system. AAV9-EF1α-DIO-mNG was used as a reporter system. (B) Representative images of the PVH immunostained for NP1 (OXT neuron). (C) Quantitative comparison of specificity to the OXT neurons. The value of the tTA-mediated labeling specificity in Figure 1 was indicated for comparison. (D) Representative images of the PVH immunostained for AVP. (E) Quantitative comparison of leaky expression of mNG in the AVP neurons. The value of the tTA-mediated leaky expression in Figure 2 was indicated for comparison. Scale bars, 200 μm. ∗∗∗*p* < 0.001; ∗∗∗∗*p* < 0.0001; n.s., not significant. Data are represented as mean ± SEM. See also Figure S4.

Since the Cre-lox recombination system is well established in neuronal experiments, most of the current AAVs are designed to use FLEX (FLip-EXcision) switches also referred to as double-floxed inverted open reading frame (DIO) systems.^47^ To assess the potential applicability of already available AAVs carrying DIO systems, we tested the compatibility of Cre-lox recombination with the principle of restricted gene expression using the TAREGET strategy instead of the Tet-Off system. We then replaced the transactivator tTA with Cre recombinase and the tTA-dependent reporter AAV with Cre-dependent reporter AAV (Figure 3A). To minimize the leaky expression of Cre recombinase, we compared two types of Cre: codon-improved Cre recombinase (iCre)^48^ and destabilized iCre (diCre), the latter of which has a protein degradation signal^49^ at the C-terminal of iCre. As a result of immunohistochemical verification, diCre-mediated labeling showed more selective localization of fluorescent signals in the NP1-positive area than iCre (Figures 3B, 3C, and S5A). However, only 32.1% of mNG-positive cells expressed NP1, suggesting that leaky diCre expression in other cell types, although non-selective labeling of AVP-positive neurons was significantly suppressed by using diCre compared to iCre (Figures 3D, 3E, and S5B). Optimization of the viral titer (1:100 dilution of diCre-driver AAV) improved the specificity of mNG expression in OXT neurons (83.1%; Figures S4C and S5D); however, its labeling efficiency decreased to 51% (Figure S5E). Therefore, the application of the TAREGET strategy to the Cre-lox system should be carefully considered, although cell-type selectivity and sparse labeling can be achieved by viral titer optimization. We conclude that the Tet-Off system is more suitable for selective and efficient gene expression in dual-AAV-based cell-type-selective labeling.

### AAV TAREGET for OXT neurons enables efficient axonal labeling of OXT neurons throughout the whole brain in mice

Next, we aimed to determine a fine circuit structure of PVH-OXT neurons in mice using AAV TAREGET-OXT. Although the wiring diagram of OXT neurons has recently been documented in detail using OXT-Cre mice,^50^^,^^51^ a non-transgenic approach will help understand the extent to which OXT circuitry is conserved across species and altered in animal models of neuropsychiatric disorders.

**Figure 4:**
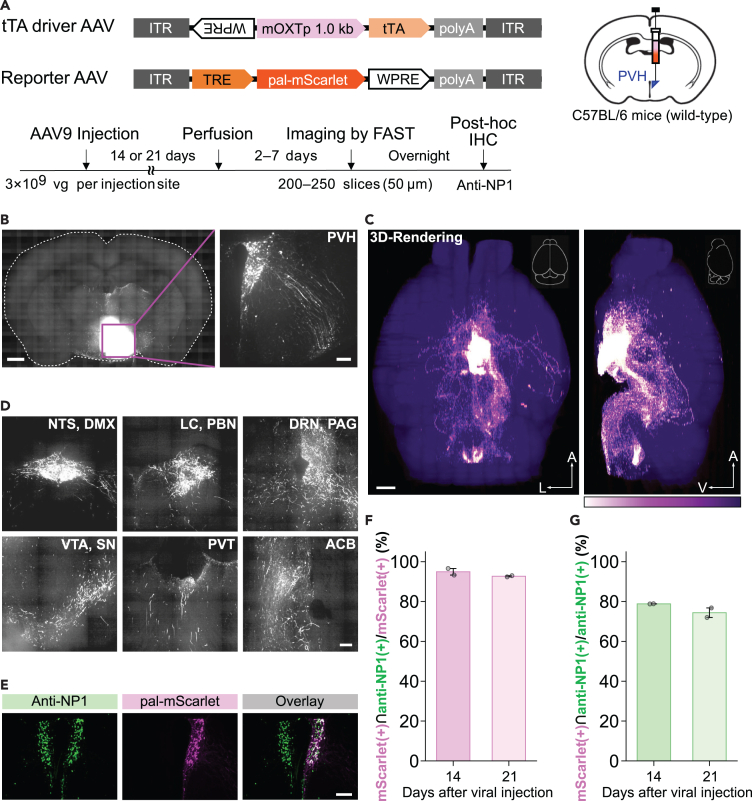
AAV TAREGET-OXT allows robust reporter gene expression to visualize axonal fibers throughout the whole brain in wild-type mice (A) Scheme of the AAV TAREGET vector constructs and experimental paradigm. AAVs were injected into the unilateral PVH region of mice at the viral dose of 3×10^9^ viral genome. Whole-brain imaging was performed using FAST, and coronal sections after FAST imaging were collected and subjected to post-hoc immunohistochemical analysis to verify the OXT neuron-selective expression. (B) Representative images of coronal section (left) and magnification of the PVH outlined with a magenta box (right). (C) The 3D-rendered images of the whole brain. The color scale represents the fluorescent intensity for the pal-mScarlet. The dim violet signal is the autofluorescence emitted from the brain parenchyma. (D) Representative images of axonal fibers in various brain regions. (E) Representative images of the post-hoc immunohistochemical analysis after FAST imaging. (F) Validation for the specificity of pal-mScarlet expression to the OXT neurons. (G) Validation for efficiency of pal-mScarlet expression in the OXT neurons. The pal-mScarlet expression was examined at 2 and 3 weeks after viral injection (E and F). NTS, nucleus tractus solitarius; DMX, dorsal motor nucleus X; LC, locus coeruleus; PBN, parabrachial nucleus; DRN, dorsal raphe nuclei; PAG, periaqueductal gray; VTA ventral tegmental area; SN, substantia nigra; PVT, paraventricular nucleus of the thalamus; ACB, nucleus accumbens; A, anterior; L lateral; V, ventral. Scale bars, 1 mm (the left panel in B and C) and 200 μm (the magnified view in B, D, and E). Data are represented as mean ± SEM. See also Figure S5.

To visualize the axonal projections of OXT neurons, we used pal-mScarlet, a bright monomeric red fluorescent protein mScarlet^52^ fused to the membrane-targeting palmitoylation domain of growth-associated protein 43 (GAP43).^44^^,^^53^^,^^54^ A cocktail of AAV-mOXT-tTA and AAV-TRE-pal-mScarlet was injected into the PVH of wild-type mice (Figures 4A and 4B). Mapping of axonal projection was conducted using a microscopic whole-brain imaging system FAST (block-FAce Serial microscopy Tomography), which we have previously developed for brain-wide structural analysis at a subcellular resolution.^55^^,^^56^ Approximately 300 cells (302 ± 49 cells) in the PVH were labeled with pal-mScarlet along the anteroposterior axis (Figure S6A), and ectopic expression was not observed throughout the brain. Extensive axonal projections were observed in the subcortical area and brain stem, whereas fewer fibers were detected in the cerebral cortex (Figures 4C and 4D), which is consistent with previous reports on OXT-Cre mice.^50^^,^^51^ To verify the cell type of pal-mScarlet-expressing cells, we also performed post-hoc immunohistochemical analysis after FAST imaging and found that the selectivity for OXT neurons was maintained at over 90% (Figures 4E–4G and S6B). These results suggest that the TAREGET strategy allows enhanced gene expression sufficient for brain-wide axonal labeling with cell-type specificity and may be applicable to a wide variety of cell-type-selective experiments in not only normal animals but animal models of brain disorders.

### Application of the TAREGET strategy in labeling dopaminergic and serotonergic neurons

Next, we applied the TAREGET strategy to genetic labeling of other cell types, dopaminergic neurons in the ventral tegmental area (VTA) and serotonergic neurons in the dorsal raphe nucleus (DRN), using the previously reported 2.6 kb length of mouse tyrosine hydroxylase promoter (mTHp)^57^ and 2.0 kb length of mouse tryptophan hydroxylase 2 promoter (mTPH2p),^58^^,^^59^ respectively. Although these promoters have already been established, we examined whether the combination of these promoters and the TAREGET strategy could maintain specificity and efficiency.

**Figure 5:**
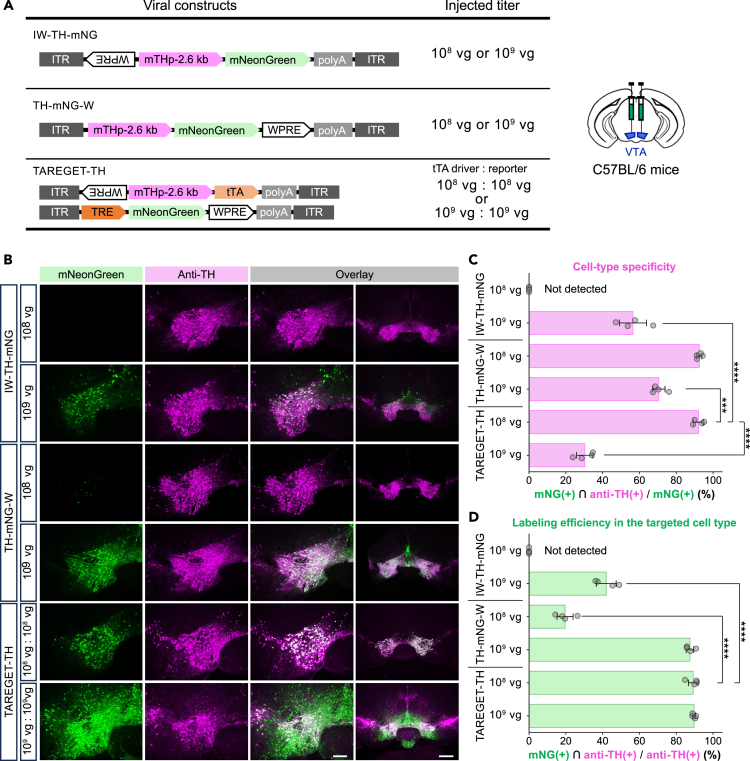
Application of the TAREGET strategy for labeling other cell types (A) Scheme of the AAV vector constructs for selective gene expression in dopaminergic neurons within the VTA using the mTH promoter. (B) Representative images of the VTA immunostained for TH (dopaminergic neurons). Each AAV group was injected at two different viral doses indicated in (A) into the VTA per side. (C) Quantification of the specificity of mNG expression for dopaminergic neurons. Percentage of mNG and TH double-positive cells among mNG-positive cells was quantified. (D) Quantification of the efficiency of mNG expression in dopaminergic neurons. Percentage of mNG and TH double-positive cells among TH-positive cells was quantified. VTA, ventral tegmental area. Scale bar, 200 μm (B, the left image in the overlays) and 500 μm (B, the right image in the overlay). ∗∗∗*p* < 0.001; *p* < 0.0001∗∗∗∗. Statistical significance is shown only for the comparisons between the optimized TAREGET-TH (10^8^ vg) and other groups to avoid redundancy. Full statistics are shown in Table S1. Data are represented as mean ± SEM. See also Figures S6–S8.

We then compared the mTHp-based TAREGET system (TAREGET-TH) and two single AAV systems carrying mTHp with 5′ inverted WPRE (IW) or 3′ WPRE (W) at two viral doses (Figure 5A). Apparent leaky expression in TH-negative cells was observed in all the viral constructs with higher viral doses (Figures 5B and 5C). The reduction in the injected viral doses dramatically decreased the number of mNG-positive cells in both single AAV systems (Figures 5D and S7). However, the TAREGET-TH at 1 × 10^8^ vg of each AAV maintained labeling efficiency in the TH-positive neurons (89.2%) and exhibited 92.1% specificity to dopaminergic neurons (Figure 5D).

Next, we evaluated the specificity and efficiency of the mTPH2p-based TAREGET system (TAREGET-TPH2) by comparing with single AAV systems carrying mTPH2p (Figure S8A). In contrast to the findings in dopaminergic neurons, the mTPH2p exhibited high specificity across examined AAV constructs, although the higher viral dose of TAREGET-TPH2 showed a slight reduction in specificity (76.1% specificity to TPH2-positive neurons) relative to other conditions (Figures S8B and S8C). However, the efficiency of labeling and the number of cells expressing mNG varied depending on the viral dose and the expression systems, with TAREGET-TPH2 showing a significant increase in efficiency compared to the single AAV systems (Figures S8D–S8F). Thus, TAREGET-TPH2 can be used for the labeling of serotonergic populations with wide coverage.

These results suggest that the TAREGET strategy is compatible with mTHp and mTPH2p and has advantages in specificity and efficiency compared to single AAV systems, although the dose of the virus should be carefully considered depending on the type of experiment.

### Cell-type-specific CRISPR-mediated gene editing using the TAREGET system

**Figure 6:**
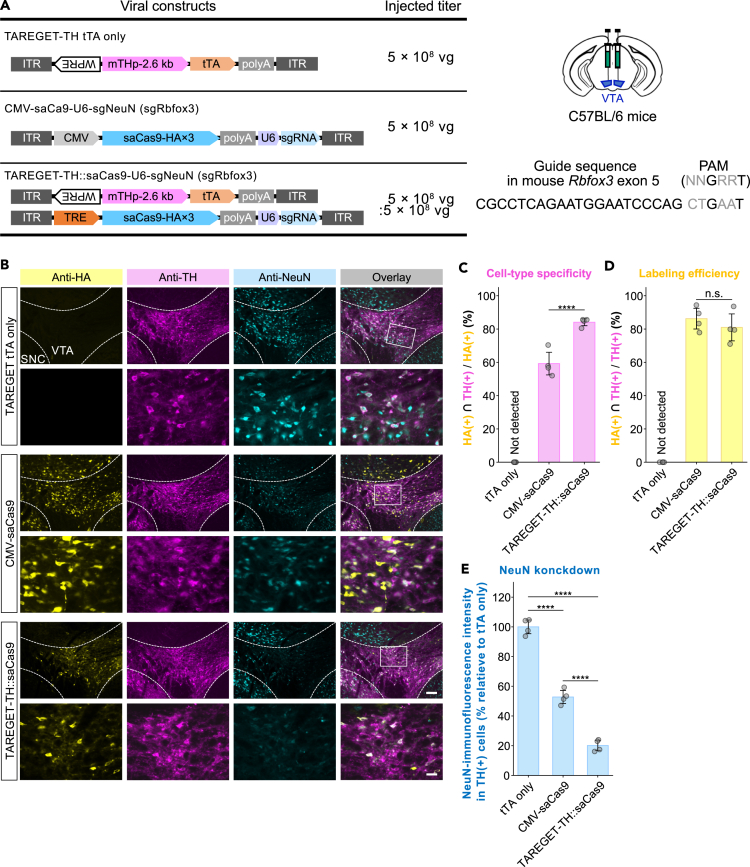
Application of the TAREGET system for cell-type-specific CRISPR-mediated gene knockdown (A) Scheme of the AAV vector constructs to evaluate knockdown of NeuN via CRISPR-mediated mutagenesis. The TAREGET-TH tTA-only group was used as a negative control that does not affect NeuN expression. The guide and protospacer adjacent motif (PAM) sequences for the mouse *Rbfox3* (NeuN) gene were indicated in the right panel. Viruses were injected into the VTA per side. (B) Representative images of the VTA immunostained for HA-tag (saCas9), TH (dopaminergic neurons), and NeuN (neuronal marker). (C) Quantification of the specificity of HA-tagged saCas9 expression to dopaminergic neurons. Percentage of HA and TH double-positive cells among HA-positive cells was quantified. (D) Quantification of the efficiency of HA-tagged saCas9 expression in dopaminergic neurons. Percentage of HA and TH double-positive cells among TH-positive cells was quantified. (E) Quantification of NeuN immunofluorescence intensity in TH-positive cells relative to the tTA-only group. Scale bar, 100 μm (B, the upper image in the overlay of TAREGET-THsaCas9) and 25 μm (B, the lower image in the overlay of TAREGET-THsaCas9). *p* < 0.0001∗∗∗∗. Data are represented as mean ± SEM.

The potential advantage of the TAREGET system is its applicability for the expression of larger genes within the limited AAV packaging capacity, owing to the compact size of the TRE promoter (approximately 320 bp) relative to other promoters (e.g., 2.6 kb of mTHp and 2.0 kb of mTPH2p). Therefore, we examined the cell-type-specific expression of *Staphylococcus aureus* Cas9 (saCas9; 3.3 kb including nuclear localization signals and HA tags)^60^ in wild-type mice and conducted gene inactivation via CRISPR-mediated mutagenesis^61^ using the TAREGET system. The expression of saCas9 was driven under the control of CMV promoter or the TAREGET-TH system and compared to the TAREGET-TH without a TRE-saCas9 vector (tTA only) (Figure 6A). To determine the efficiency of CRISPR-mediated knockdown at protein levels in the targeted cell type, we generated a single guide RNA (sgRNA) targeting the *Rbfox3* (NeuN) gene, which is a neuron-specific protein localized in nuclei and perinuclear cytoplasm^62^ (Figure 6A).

Specific and efficient expression of HA-tagged saCas9 in the TH-positive population was achieved using the TAREGET-TH system (Figures 6B–6D). The CMV-saCas9 vector resulted in a reduction of NeuN immunofluorescence in both the TH-positive and TH-negative area consistent with its non-specific expression of saCas9, whereas TAREGET-TH-mediated saCas9 expression attenuated the NeuN immunofluorescence in the TH-positive cells specifically (Figure 6B). The fluorescence intensity of NeuN in TH-positive cells was significantly decreased by saCas9-expressing vectors compared to the tTA-only negative control (Figure 6E), suggesting the efficient knockdown of NeuN via CRISPR-mediated mutagenesis. These results indicate that cell-type-specific genome editing in wild-type mice can be achieved using the TAREGET system.

## Discussion

In the present study, we propose an AAV system for cell-type-selective gene delivery in wild-type animals, named TAREGET. The main principle of AAV TAREGET is tight but efficient control of gene expression in the targeted cell population using an inverted WPRE, a cell-type-selective promoter, and a Tet-Off system. The use of binary expression systems, such as the Cre-lox and Tet-Off systems, has become widespread and prevalent in transgenic animals,^63^ but these are less established in viral-genetic approaches carrying tTA or Cre driver genes. The limited utility of AAV vectors is partially due to non-specific labeling caused by leaky background expression of driver genes. In binary approaches, reporter gene expression can be induced by the leaky and weak expression of driver genes, which is attributed to the insufficient specificity of the cell-type-targeting promoter, non-specific ITR promoter activity,^37^^,^^38^ or a high copy number of infected AAV vectors.^19^ Insufficient regulation of driver gene expression can also lead to toxic effects, as observed with extremely high levels of transgene expression induced by the tTA-WPRE combination (Figure S1). Overall, our results suggest that the TAREGET system can be used for achieving appropriate levels of transgene expression with cell-type specificity, labeling efficiency, and less cytotoxicity. Furthermore, the TAREGET strategy allowed cell-type specific and efficient expression in multiple cell types: oxytocinergic, dopaminergic, and serotonergic neurons. These results suggest that the TAREGET system has potential for broader application in other cell-type-selective promoters in AAV vectors.

An additional advantage of the dual AAV system is its greater packaging capacity compared to a single AAV system. The packaging capacity of AAV, typically 4.7 kb, limits the inclusion of large cell-type-specific promoters along with large transgenes. In contrast, the compactness of the TRE promoter in the Tet-Off-based dual AAV system allows for the use of a large part of the limited packaging capacity for the transgene, as previously applied to express saCas9 depending on neuronal activation.^64^ Although the advantage of increased transgene capacity is a common feature among AAVs equipped with the TRE promoter, the TAREGET system enhances cell-type specificity and ensures extensive coverage within targeted cell types for long transgenes. Thus, the TEREGET system not only refines specificity for targeted cell types but also expands the variety of experiments for cell-type-selective manipulation in neuroscience, including gene editing. Furthermore, given that the TAREGET system is compatible with expression logics using Cre and Flp recombinases (e.g., TRE-DIO-mNG cassette), it enables molecular and cellular interventions in complex cell subtypes, including genetically and/or anatomically defined subpopulations.

Experimental conditions, such as AAV capsid serotypes and application to reporter mouse lines should be carefully considered; different conditions may result in different outcomes. Studies of high-quality single-cell ATAC sequencing have identified cell-type- or subclass-specific enhancers,^19^^,^^20^ and one of these studies showed that some cell-type-specific enhancer AAVs carrying tTA resulted in sparse labeling.^19^ Although the target cell types differed across studies, the labeling efficiency may be influenced by methodological diversities in the AAV capsid serotypes (e.g., serotype 9 vs. PHP.eB), routes of viral administration (e.g., stereotaxic injection vs. intravenous administration), and reporter systems (e.g., reporter AAV vs. reporter mouse line). This concern is partially supported by a study showing that the use of different serotypes results in different specificities of labeling in noradrenergic neurons, probably due to differences in transduction efficiency.^65^ Indeed, TAREGET AAVs for serotonergic and dopaminergic neurons require optimization of transduction efficiency by adjusting viral doses. Thus, we should note that the selectivity and completeness of labeling may depend not only on promoter and regulatory elements of the recombinant AAV genome, but also on the infection efficiency and reporter systems employed. Conversely, the TAREGET strategy may provide different results with improved specificity and efficiency and has the potential to renew the previously identified enhancers or promoters used in conventional AAV systems.

The PVH and supraoptic nucleus (SON) are the major sources of OXT, not only for peripheral release but also for the central nervous system.^66^ OXT is released from the axonal and somatodendritic processes^67^ and modulates neuronal activity in target brain regions via OXT receptor.^68^ The distribution of OXT receptors and OXT-neuronal axons has been reported to have discrepancies, such that the OXT receptor is expressed in neurons in a wide range of cortical regions, but few axons from OXT neurons in the PVT and SON are detected in the cortex.^50^ In the present study, we applied the AAV TAREGET-OXT to brain-wide axonal mapping to examine whether we could obtain results similar to those of previous studies using OXT-Cre mice and whole-brain imaging. Consistent with previous studies, dense axonal projections from the PVH were observed mainly in the hypothalamus, midbrain, and hindbrain. The OXT projection to the cortical area was quite sparse, although we employed a membrane-tagged fluorescent protein for better axonal transports^44^^,^^54^ and higher-resolution imaging conditions (1 × 1 μm for lateral resolution and 5 μm for axial resolution) for comprehensive mapping of axons throughout the entire cortex. These results may support the idea from previous studies that OXT can act on OXT receptor-expressing cortical neurons via the cerebrospinal fluid.^35^ Dense OXT projections adjacent to the ventricles (dorsal and ventral parts of the third ventricle, fourth ventricle, and cerebral aqueduct; Figure 4D) may support the release of OXT into the cerebrospinal fluid, as suggested in a previous report.^50^ These alternative tools for cell-type-specific approaches allow the fine structural analysis of specific cell types in a variety of mouse models without using Cre-driver mouse lines.

### Limitations of the study

In the present study, we optimized the combination of regulatory components in recombinant AAV vectors to establish selective and efficient transduction in targeted neuronal populations. The main limitations of the TAREGET system are (1) the necessity to use two distinct vectors in a precise ratio, and (2) the necessity of inherently cell-type-specific promoters. Therefore, pilot experiments are required to achieve specific and efficient expression, and those can limit the applicability and translatability of the TAREGET system to other species. Despite these limitations, our study empirically and quantitatively demonstrated that cell-type specificity and efficiency of gene expression can be enhanced by modifying gene regulatory elements other than promoters. These methodologies and the potential characteristics of AAV vectors can be essential information for guiding reproducible experiments and their translational applications.

For practical concerns for the use in basic animal research, we have not examined the cell-type selectivity of TAREGET AAVs in unintended other brain regions, such as the cortical or thalamic areas, where oxytocinergic, serotonergic, and dopaminergic neurons are not located. Therefore, we could not exclude the potential ectopic expression when TAREGET AAVs were applied to unintended brain regions by widespread transduction, such as AAV2-*retro*-mediated retrograde transduction^69^ and PHP.eB capsid-mediated brain-wide transduction.^57^^,^^70^ Ectopic expression can be induced by the TRE promoter itself in a doxycycline-independent manner.^71^ Some genes contain transcription factor recognition sequences in their protein coding sequence themselves,^72^ and those may enhance tTA-independent expression. These factors in the TRE promoter system can affect the functionality of AAV TAREGETs. Therefore, users must carefully examine cell-type specificity and efficiency when the TAREGET system is applied to expressing other genes of interest that we have not examined.

## STAR★Methods

### Key resources table

**Table:** 

REAGENT or RESOURCE	SOURCE	IDENTIFIER
**Antibodies**		

Anti-Neurophysin 1 Antibody, clone PS 38	Merck	Cat# MABN844
Recombinant Anti-Vasopressin antibody	Abcam	Cat# ab213708
Rabbit Cleaved Caspase-3 (Asp175) Antibody	Cell Signaling Technology	Cat# 9661S RRID:AB_2341188
Rabbit Anti-Tryptophan Hydroxylase 2 Antibody	Novus Biologicals	Cat# NB100-74555, RRID:AB_1049988
Chicken Anti-Tyrosine Hydroxylase Antibody	Abcam	Cat# ab76442, RRID:AB_1524535
Mouse Anti-HA-tag mAb	MBL	Cat# M180-3; RRID:AB_10951811
Rabbit Anti-NeuN antibody	Abcam	Cat# ab177487; RRID:AB_2532109
Goat Anti-Mouse IgG H&L (Alexa Fluor® 488)	Abcam	Cat# ab150113, RRID:AB_2576208
Goat Anti-Mouse IgG H&L (Alexa Fluor® 568)	Abcam	Cat# ab175473, RRID:AB_2895153
Goat Anti-Rabbit IgG H&L (Alexa Fluor® 568)	Abcam	Cat# ab175471, RRID:AB_2576207
Goat Anti-Chicken IgY H&L (Alexa Fluor® 568)	Abcam	Cat# ab175477; RRID:AB_3076392
Goat Anti-Mouse IgG H&L (Alexa Fluor® 647)	Abcam	Cat# ab150115; RRID:AB_2687948
Goat Anti-Chicken IgY H&L (Alexa Fluor® 647)	Abcam	Cat# ab150171; RRID:AB_2921318

**Bacterial and virus strains**		

DH5α competent cells	Takara Bio Inc.	Cat# 9057
AAV9-mOXTp-1.0 kb-tTA-WPRE-pA	This paper	N/A
AAV9-mOXTp-1.0 kb-tTA-pA	This paper	N/A
AAV9-IW-mOXTp-1.0 kb-tTA-pA (TAREGET-OXT)	This paper	N/A
AAV9-mOXTp-1.0 kb-mNG-WPRE-pA	This paper	N/A
AAV9-mOXTp-1.0 kb-mNG-pA	This paper	N/A
AAV9-IW-mOXTp-1.0 kb-mNG-pA	This paper	N/A
AAV9-mTHp-2.6 kb-mNG-WPRE-pA	This paper	N/A
AAV9-IW-mTHp-2.6 kb-mNG-pA	This paper	N/A
AAV9-IW-mTHp-2.6 kb-tTA (TAREGET-TH)	This paper	N/A
AAV9-mTPH2p-2.0 kb-mNG-WPRE-pA	This paper	N/A
AAV9-IW-mTPH2p-2.0 kb-mNG-pA	This paper	N/A
AAV9-IW-mTPH2p-2.0 kb-tTA (TAREGET-TPH2)	This paper	N/A
AAV9-TRE-mNG-WPRE-pA	This paper	N/A
AAV9-TRE-mNG-pA	This paper	N/A
AAV9-IW-mOXTp-2.6 kb-tTA-pA	This paper	N/A
AAV9-IW-mOXTp-1.0 kb-iCre-pA	This paper	N/A
AAV9-IW-mOXTp-1.0 kb-diCre-pA	This paper	N/A
AAV9-EF1α-DIO-mNG-WPRE-pA	This paper	N/A
AAV9-TRE-pal-mScarlet-WPRE-pA	Hirato et al., 2024^73^	N/A
AAV9-CMV-saCas9-pA-U6-sgNeuN(Rbfox3)	This paper	N/A
AAV9-TRE-saCas9-pA-U6-sgNeuN(Rbfox3)	This paper	N/A

**Chemicals, peptides, and recombinant proteins**		

In-Fusion HD Cloning Kit	Takara	Cat# 639650
DNA Ligation Kit	Takara	Cat# 6023
Phusion High-Fidelity DNA polymerase	Thermo Fisher	F530L
PEI MAX	Polyscience Inc.	Cat# 24765
DMEM, high glucose, GlutaMAX™ Supplement	Thermo Fisher	Cat # 10566024
Benzonase® Nuclease	Millipore	Cat# E1014-25KU

**Critical commercial assays**		

GoTaq® qPCR Master Mix	Promega	Cat# A6001/2

**Experimental models: Cell lines**		

Lenti-X™ 293T cell line	Clontech	Cat# 632180

**Experimental models: Organisms/strains**		

C57BL/6NCrSlc	Japan SLC,Inc.	MGI: 5295404
C57BL/6JJmsSlc	Japan SLC,Inc.	MGI: 5488963

**Recombinant DNA**		

pAAV-mOXTp-1.0 kb-tTA-WPRE-pA	This paper	N/A
pAAV-mOXTp-1.0 kb-tTA-pA	This paper	N/A
pAAV-IW-mOXTp-1.0 kb-tTA-pA (TAREGET-OXT)	This paper	N/A
pAAV-mOXTp-1.0 kb-mNG-WPRE-pA	This paper	N/A
pAAV-mOXTp-1.0 kb-mNG-pA	This paper	N/A
pAAV-IWmOXTp-1.0 kb-mNG-pA	This paper	N/A
pAAV-mTHp-2.6 kb-mNG-WPRE-pA	This paper	N/A
pAAV-IW-mTHp-2.6 kb-mNG-pA	This paper	N/A
pAAV-IW-mTHp-2.6 kb-tTA (TAREGET-TH)	This paper	N/A
pAAV-mTPH2p-2.0 kb-mNG-WPRE-pA	This paper	N/A
pAAV-IW-mTPH2p-2.0 kb-mNG-pA	This paper	N/A
pAAV-IW-mTPH2p-2.0 kb-tTA (TAREGET-TPH2)	This paper	N/A
pAAV-TRE-mNG-WPRE-pA	Chan et al., 2017^57^	Addgene, #99131 RRID: Addgene_99131
pAAV-TRE-mNG-pA	This paper	N/A
pAAV-IW-mOXTp-2.6 kb-tTA-pA	This paper	N/A
pAAV-IW-mOXTp-1.0 kb-iCre-pA	This paper	N/A
pAAV-IW-mOXTp-1.0 kb-diCre-pA	This paper	N/A
pAAV-EF1α-DIO-mNG-WPRE-pA	This paper	N/A
pAAV-TRE-pal-mScarlet-WPRE-pA	Hirato et al., 2024^73^	N/A
pAAV-CMV-saCas9-pA-U6-BsaI-sgRNA	https://www.addgene.org/61591/	Addgene, #61591 RRID:Addgene_61591
pAAV-CMV-saCas9-pA-U6-sgNeuN(Rbfox3)	This paper	N/A
pAAV-TRE-saCas9-pA-U6-sgNeuN(Rbfox3)	This paper	N/A
pAAV-mOXT-hM3Dq-mCherry	Peñagarikano et al., 2015^29^	Addgene, #70717 RRID: Addgene_70717
pAAV-TRE-DIO-FlpO	Lin et al., 2018^74^	Addgene, #118027 RRID: Addgene_118027
pAAV-EF1-DIO-hM3Dq-mCherry	https://www.addgene.org/50460/	Addgene, #50460 RRID: Addgene_50460
pAAV-CAG-fDIO-mNeonGreen	Chan et al. 2017^57^	Addgene, # 99133 RRID: Addgene_99133
pAAV-CaMKIIa-mScarlet	Marshel et al., 2019^75^	Addgene, #131000 RRID: Addgene_131000
pHelper-AAV	Cell Biolabs	Part# 340202 in Cat# VPK-400-DJ
pAAV2/9n	https://www.addgene.org/112865/	Addgene, #112865 RRID: Addgene_112865

**Software and algorithms**		

Fiji for Windows	Schindelin et al., 2012^76^	RRID: SCR_002285 https://fiji.sc/
R v.4.2.2.	R project	https://www.r-project.org/
Rstudio Desktop	Rstudio	RStudio Desktop - Posit
ApE (A plasmid Editor)	Davis et al., 2022^77^	RRID: SCR_014266 https://jorgensen.biology.utah.edu/wayned/ape/
Imaris	BitPlane	N/A
FASTicher (whole-brain image stitching pipeline)	Seiriki et al., 2019^56^	N/A

**Other**		

Microinjection Syringe Pump with Smartouch Controller	WPI	UMP3T-1
Neuros Syringe	HAMILTON	Cat# 65460-05 Cat# 65460-06

### Resource availability

#### Lead contact

Further information and requests for resources and reagents should be directed to and will be fulfilled by the lead contact, Kaoru Seiriki (seiriki@phs.osaka-u.ac.jp).

#### Materials availability

Plasmids generated in this study will be available by the lead contact upon request.

#### Data and code availability

• All data reported in this paper will be shared by the lead contact upon request.
• This paper does not report original code.
• Any additional information required to reanalyze the data reported in this paper is available from the lead contact upon request.

### Experimental model and study participant details

#### Animals

All animal care and handling procedures were approved by the Animal Care and Use Committee of Osaka University (approval number R02-8-7). All efforts were made to minimize the number of animals used. Male C57BL/6N or C57BL/6J mice (SLC, Shizuoka, Japan) aged 7–14 weeks were used throughout this study. The mice were maintained in group housing (three–six mice per cage), except for singly housed mice. They were kept on a 12-h light-dark cycle (lights on at 8:00 a.m.) with controlled room temperature and humidity. Water and food (CMF, Oriental Yeast, Osaka, Japan) were available *ad libitum*.

#### Cell lines

Lenti-X 293T cells (632180, Clontech) were used to produce the AAV. Cells were cultured in Dulbecco’s modified Eagle’s medium with high glucose and GlutaMAX supplement (DMEM, high glucose, GlutaMAX Supplement; 10566024, Thermo Fisher Scientific) containing 10% fetal bovine serum (Sigma-Aldrich) at 37°C with 5% CO_2_.

### Method details

#### Plasmid construction

A plasmid editor (ApE)^77^ was used for designing the plasmids. For AAV TAREGET-OXT plasmid construction, the tTA advanced and SV40 polyadenylation signal sequences were cloned by PCR amplification from the pTet-Off Advanced vector (631070, Clontech) and inserted into a linearized pAAV-TRE-DIO-FlpO^74^ (Addgene #118027) backbone (digested with HindIII and MluI) using an In-Fusion cloning system (639650, Clontech). The residual hGH polyadenylation signal in the plasmid backbone was removed by digestion with BstEII and BglII, followed by blunt endings and self-ligation. The resulting plasmid had a promoterless TAREGET backbone, pAAV-inverted WPRE (IW)-tTA-pA. The 1.0 kb^29^ and 2.6 kb^32^ lengths of mOXTp were cloned by PCR amplification using pAAV-mOXT-hM3Dq-mCherry^29^ (#70717, Addgene) and C57BL/6J mouse genomic DNA, respectively, and inserted into the TAREGET backbone using the In-Fusion system. For TAREGET-TPH2 and TAREGET-TH plasmid construction, the 2.0 kb length of mTPH2p^58^^,^^59^ and the 2.6 kb length of mTHp^57^ were cloned by PCR amplification using C57BL/6J mouse genome DNA and inserted into the TAREGET backbone in the same way as the TAREGET-OXT. The other plasmids shown in Figure 1 (plasmids for OXT neurons), Figure 5 (plasmids for dopaminergic neurons), and Figure S8 (plasmids for serotonergic neurons) were prepared from pAAV-mOXT-hM3Dq-mCherry, pAAV-TRE-mNeonGreen^57^ (#99131, Addgene), and plasmids for TAREGET AAVs carrying mOXTp, mTHp, and mTPH2p, using basic subcloning techniques. For Cre-driver plasmid construction, tTA in the AAV TAREGET-OXT (pAAV-IW-mOXT-tTA) was removed using EcoRI and BamHI, and iCre or iCre together with the degradation signal CL1-PEST, which was cloned by PCR from the pAAV-Fos-dER^T2^CreER^T2^ plasmid,^78^ was inserted using the In-Fusion system. To construct pAAV-EF1-DIO-mNeonGreen, pAAV-EF1-DIO-hM3Dq-mCherry (#50460, Addgene) and pAAV-CAG-fDIO-mNeonGreen^57^ (#99133, Addgene) were digested using AscI and NheI, and the mNeonGreen DNA fragment was inserted into the linearized pAAV-EF1-DIO vector. To construct pAAV-TRE-pal-mScarlet-WPRE, mScarlet was cloned and fused to the C-terminus of the GAP43 palmitoylation domain by PCR amplification using pAAV-CaMKIIa-mScarlet^75^ (#131000, Addgene) and a primer with an additional sequence encoding the GAP43 palmitoylation domain as previously described.^73^ The pal-mScarlet PCR amplicon was inserted into the linearized pAAV-TRE vector (pAAV-TRE-mNeonGreen digested with KpnI and EcoRI) using the In-Fusion system. To construct pAAV-CMV-saCas9-U6-sgNeuN, the guide sequence for sgRNA targeting mouse NeuN (*Rbfox3*), 5′- CGCCTCAGAATGGAATCCCAG-3′ was designed using the Benchling CRISPR Guide RNA Design tool (https://www.benchling.com/crispr). The pAAV-CMV-saCas9-U6-BsaI-sgRNA (#61591, Addgene) was digested with BsaI, and synthesized oligonucleotides (the guide sequence with complementary sequences to the overhangs generated by BsaI digestion) were inserted by ligation. To further construct pAAV-TRE-saCas9-U6-sgNeuN, the mNeonGreen gene and WPRE sequence in the pAAV-TRE-mNeonGreen was replaced with saCas9-U6-sgNeuN in the pAAV-CMV-saCas9-U6-sgNeuN via restriction enzyme digestion with NcoI and PvuI followed by ligation.

#### Viral vector production and purification

AAV vectors were produced using the helper-free triple transfection procedure, as described previously^70^ with minor modifications. Briefly, an AAV transgene plasmid was transfected into Lenti-X 293T cells using polyethyleneimine (PEI MAX; 24765, Polyscience Inc.) with a pAAV2/9n plasmid (#112865, Addgene), which supplied AAV2 replication proteins and AAV9 capsid proteins, and a pHelper plasmid, which supplied the adenovirus gene products required for AAV production. Cells were harvested 72 h after transfection and suspended in a buffer containing 100 mM Tris (pH 7.6), 100 mM MgCl2, and 500 mM NaCl. The cell suspension was subjected to three freeze-thaw cycles and treated with ≥250 U/μL of Benzonase nuclease (Sigma-Aldrich, St Louis, MO) at 37°C for at least 40 min. The suspension was centrifuged at 3,000 × *g* for 15 min at 4°C, and the supernatant was loaded onto an iodixanol step gradient (15, 25, 40, and 60%; Optiprep, Cosmo Bio) and centrifuged at 278,400 × *g* for 105 min at 18°C. After centrifugation, the 40/60% interface and the 40% layer containing AAV vectors were collected and replaced with D-PBS containing 0.001% (v/v) Pluronic F-68 via ultrafiltration using an Amicon Ultra-15 centrifugal filter (100-kDa cutoff; UFC910024, Millipore) to concentrate the AAV vectors. The titer of AAV was quantified using a quantitative real-time PCR with GoTaq qPCR Master Mix (Cat# A6001, Promega), with a linearized AAV genome plasmid serving as a standard.

#### Stereotaxic surgery

Mice were deeply anesthetized by intraperitoneal injection of an anesthetic cocktail containing 0.75 mg/kg medetomidine (Nippon Zenyaku Kogyo, Fukushima, Japan), 4 mg/kg midazolam (Sandoz Pharma, Basel, Switzerland), and 5 mg/kg butorphanol (Meiji Seika Pharma, Tokyo, Japan) and placed in a stereotaxic instrument (RWD Life Science Co., LTD, San Diego, CA). The AAV vectors were injected into the bilateral or unilateral PVH (anteroposterior (AP) −0.7 mm, mediolateral (ML) ±0.3 mm, dorsoventral (DV) −5.0 mm to −5.2 mm from the bregma), the DRN (AP −4.6 mm, ML 0.0 mm, DV −3.3 mm from the bregma with 20°-angled injection), and the VTA (AP −3.6 mm, ML ±0.6 mm, DV −4.6 mm from the bregma) using a Neuros Syringe with a 33-gauge needle (Hamilton, Reno, NV) and an UltraMicroPomp3 with SMARTouch Controller (World Precision Instruments) at a rate of 100 nL/min. For TAREGET-OXT AAVs, each AAV set was injected at doses of 1 × 10^9^ and 3 × 10^9^ vg (viral genomes) per side, as shown in Figures 1, 2, 3, and 4, respectively. For AAVs carrying the mTHp and mTPH2p, each single AAV was injected at two doses: 1 × 10^8^ vg and 1 × 10^9^ vg, and the TAREGET-TH AAV cocktail (tTA driver AAV: TRE reporter AAV) was injected at two ratios: 1 × 10^9^ vg with 1 × 10^9^ vg, and 1 × 10^8^ vg with 1 × 10^8^ vg per side. For TAREGET-TPH2, the AAV cocktail (tTA driver AAV: TRE reporter AAV) was injected at two ratios: 1 × 10^9^ vg with 1 × 10^9^ vg, and 1 × 10^7^ vg with 1 × 10^9^ vg. The needle was kept in place for 3 min before being slowly removed to avoid backflow. Following stereotaxic surgery, the mice received an intraperitoneal injection of atipamezole (7.5 mg/kg; Nippon Zenyaku Kogyo) and gentamicin (10 mg/kg; Sigma-Aldrich) immediately. One day after the surgery, an intraperitoneal injection of buprenorphine (0.1 mg/kg; Otsuka Pharma, Tokyo, Japan) was administered to relieve pain.

#### Tissue preparation

Mice were deeply anesthetized by intraperitoneal injection of an anesthetic cocktail containing medetomidine (0.75 mg/kg), midazolam (4 mg/kg), and butorphanol (5 mg/kg). Anesthetized mice were transcardially perfused with saline followed by 4% paraformaldehyde (Nacalai Tesque, Kyoto, Japan) dissolved in phosphate-buffered saline (PBS). Brain tissues were excised and immersed in a 4% paraformaldehyde solution until use. For immunohistochemical analysis, fixed brains were cryoprotected in 20% (w/v) sucrose dissolved in PBS for two days, embedded in OCT compound (Sakura Finetek) and quickly frozen on liquid nitrogen. For whole-brain imaging, fixed brains were embedded in 4% (w/v) agarose gel.

#### Immunohistochemical analysis

Brain tissues were sliced into 25-μm-thick coronal sections using a cryostat. Serial sections were alternately and separately collected into two groups (e.g., 20–30 sections from one brain per group for the PVH). For immunohistochemical analysis of the PVH, one group of sections was used for immunostaining for NP1, and the other was used for immunostaining for AVP, as necessary. For the immunohistochemical analysis of the DRN and VTA, only one group of sections was used for immunostaining. In the post-hoc immunohistochemical analysis after whole-brain imaging in Figure 4, the 50-μm-thick coronal sections after FAST imaging were collected (see also Whole-brain imaging method section). The sections were subjected to conventional free-floating staining. Briefly, sections were incubated with 1% bovine serum albumin (Nacalai Tesque) dissolved in PBS containing 0.3% (v/v) Triton X-100 (Nacalai Tesque) for 1 h at room temperature for blocking and permeabilization.

To determine the cell type expressing fluorescent proteins, sections were stained by following procedures. For the primary antibody reaction, sections were incubated with mouse anti-NP1 monoclonal antibody (1:1,000 dilution; cat# MABN844, Merck Millipore), rabbit anti-AVP polyclonal antibody (1:1,000 dilution; cat# ab213708, abcam), rabbit anti-TPH2 polyclonal antibody (1:1000 dilution; cat# NB100-74555, Novus Biologicals) or chicken anti-TH polyclonal antibody (1:1000 dilution; cat# ab76442, abcam) overnight at room temperature. For the secondary antibody reaction, the sections were incubated with Alexa 568-conjugated goat anti-mouse or anti-rabbit immunoglobulin G (IgG), Alexa 568-conjugated goat anti-chicken immunoglobulin Y (IgY), or Alexa 488-conjugated goat anti-mouse IgG antibodies (1:1,000 dilution; cat# ab175473, ab175471, ab175477 and ab150113, respectively, abcam) for 2 h at room temperature. For the evaluation of cell death induction, rabbit anti-cleaved caspase-3 antibody (1:400 dilution; cat# 9661S, Cell Signaling Technology) and Alexa 568-conjugated goat anti-rabbit IgG antibody (1:1,000 dilution) were used with mouse anti-NP1 and Alexa 647-conjugated goat anti-mouse IgG antibodies (cat# ab150115 for secondary antibody, abcam). For the knockdown experiment using AAVs carrying the saCas9 gene, sections were stained as follows. For the primary antibody reaction, sections were incubated with mouse anti-HA-tag monoclonal antibody (1:1,000 dilution; cat# M180-3, MBL, Tokyo, Japan), rabbit anti-NeuN monoclonal antibody (1:500 dilution; cat# ab177487, abcam), and chicken anti-TH polyclonal antibody (1:1,000 dilution; cat# ab76442, abcam) overnight at room temperature. For the secondary antibody reaction, sections were incubated with Alexa 488-conjugated goat anti-mouse IgG, Alexa 568-conjugated goat anti-rabbit IgG, and Alexa 647-conjugated goat anti-chicken IgY antibodies (1:1,000 dilution; ab150113, ab175471, and ab150171, respectively, abcam) for 2 h at room temperature. Tissue sections with following AP coordinates were imaged for quantification of fluorescence-positive cells using a fluorescence microscope (BZ-X810, Keyence): AP −1.2 mm to 0 mm for the PVH; AP −4.7 mm to −4.0 mm for the DRN; and AP −3.7 mm to −3.3 mm for the VTA. The number of fluorescence-positive cells in the field of view (FOV; 1.08 mm × 1.44 mm) including the PVH, DRN, and VTA was manually counted using ImageJ/FIJI.^76^ Specificity, efficiency, and leakage were calculated for each injection site, and cells in the bilateral PVH and VTA were counted separately. The immunofluorescence intensity of NeuN in dopaminergic neurons was quantified using ImageJ/FIJI as follows: (1) the region of interest (ROI) mask for TH-positive cells were generated using background subtraction and binarization by brightness-thresholding, and (2) the fluorescence intensity of immunolabeled NeuN per TH-positive ROI area was measured.

#### Whole-brain imaging and data processing

Whole-brain imaging was performed using a high-speed serial-section imaging system, FAST, which was developed in previous studies.^55^^,^^56^ Briefly, brain tissue blocks embedded in a 4% agarose gel were placed in the sample chamber of the FAST system. Brain section images were acquired as a mosaic of fields of view with 20% overlap in the x-y plane and 25-μm overlap in the z-direction, with a resolution of 1.0 μm × 1.0 μm × 5.0 μm. We acquired images with two color channels: red fluorescence excited by a 561 nm laser and green autofluorescence excited by a 488 nm laser. The x-y plane section images were reconstructed by overlapping the alignment of consecutive sections using a previously reported stitching program, FASTicher,^56^ with minor modifications in Python 3.8. Reconstructed serial section images were downsized to 5.0 μm × 5.0 μm × 50 μm and exported to Imaris software (Bitplane) for three-dimensional volume rendering.

### Quantification and statistical analysis

All data are presented as the mean ± SEM. Statistical analyses were conducted on R using the following functions: var.test(), t.test(), bartlett.test(), oneway.test(), and pairwise.t.test(). For two-group comparisons, the F-test was used to assess the equality of variances, and the unpaired Student’s t test or Welch’s t-test was used, depending on the equality of variances. For multiple comparisons, equality of variances was examined by the Bartlett test, and statistical significance was evaluated by one-way ANOVA followed by the Holm-Bonferroni test or Welch’s ANOVA followed by Welch’s t-test with Holm correction depending on the equality of variances. Statistical information, including the sample size, is indicated in the figure legends. Statistical significance was set at *p* < 0.05∗, *p* < 0.01∗∗, *p* < 0.001∗∗∗, and *p* < 0.0001∗∗∗∗.
