# Supplementary material for: Optimization of AAV vectors for transactivator-regulated enhanced gene expression within targeted neuronal populations
Source: iScience. 2024 May 3;27(6):109878. doi: 10.1016/j.isci.2024.109878 (PMC11126825; doi:10.1016/j.isci.2024.109878)
Supplement: Document S1. Figures S1–S8 and Table S1 [file mmc1.pdf]

iScience, Volume *27*

Supplemental information

Optimization of AAV vectors for transactivator-regulated enhanced gene expression within targeted neuronal populations

Leo Kojima, Kaoru Seiriki, Hiroki Rokujo, Takanobu Nakazawa, Atsushi Kasai, and Hitoshi Hashimoto

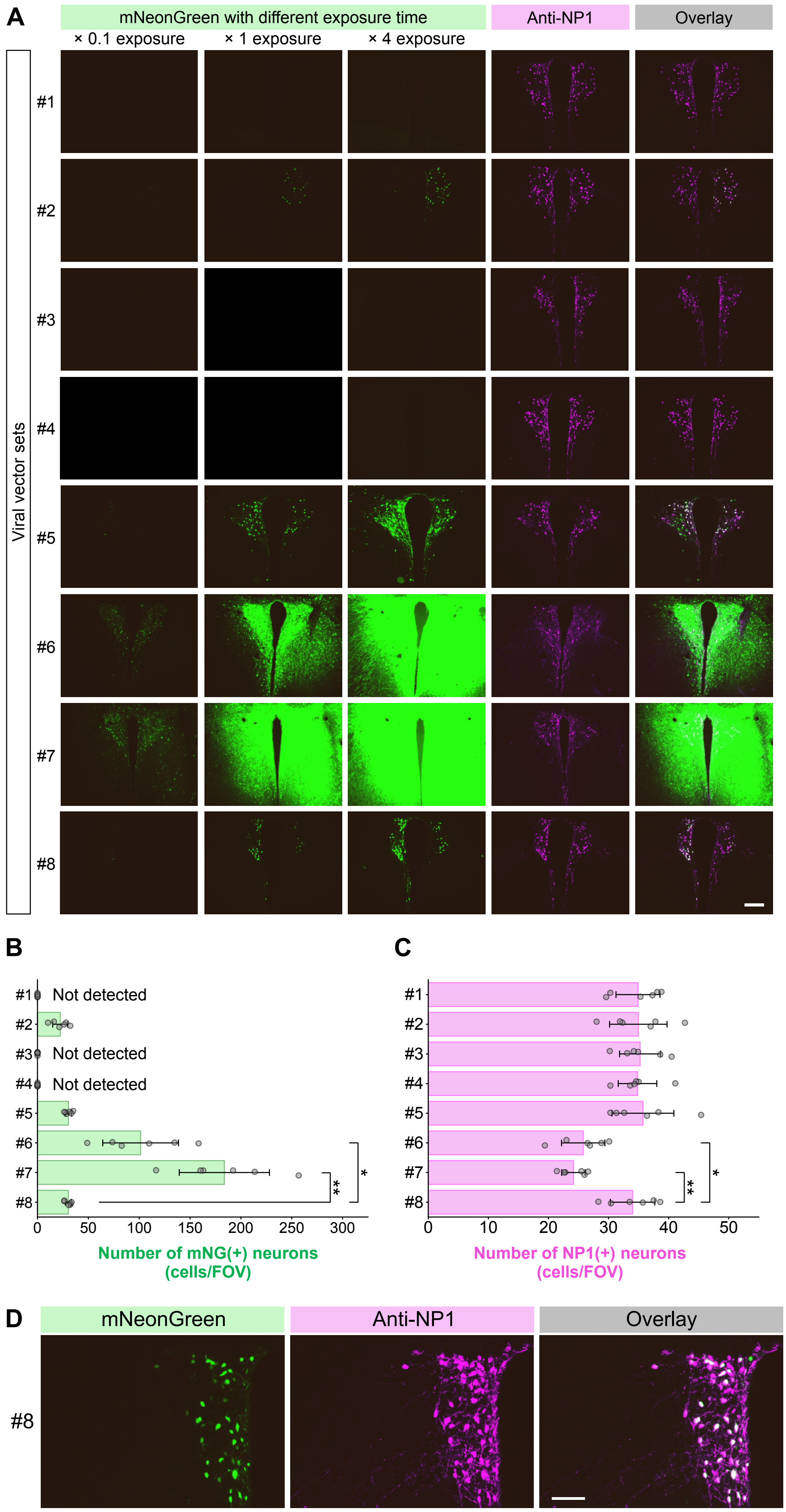

Figure S1. (legend on next page)

Figure S1. Specificity and efficiency of AAV gene delivery are affected by non-promoter regulatory components (related to Figure 1)

(A) Representative images showing the signals of mNG (green), NP1-immunofluorescence (magenta), and their overlay in the sections −1.0 to −0.8 mm anteroposterior from bregma. The numbers #1–8 indicated in the row labels represent the number of AAV set in Figure 1. The mNG channels were acquired at three different exposure times (×0.1, ×1, and ×4) represented on the column labels. The images at ×1 exposure time are acquired at the same exposure time with images in Figure 1. (B) The number of mNG-positive neurons per field of view (FOV). (C) The number of NP1-positive neurons per FOV. For B and C, the cell numbers were calculated as a mean of cells per ROI in the single coronal section. (D) Representative magnified images of the PVH using the AAV set #8. *$p < 0.05$; **$p < 0.01$. Statistical significance is shown only for the comparisons between AAV set #8 (TAREGET-OXT) and other groups to avoid redundancy. Full statistics are shown in Table S1. Data are represented as mean ± SEM.

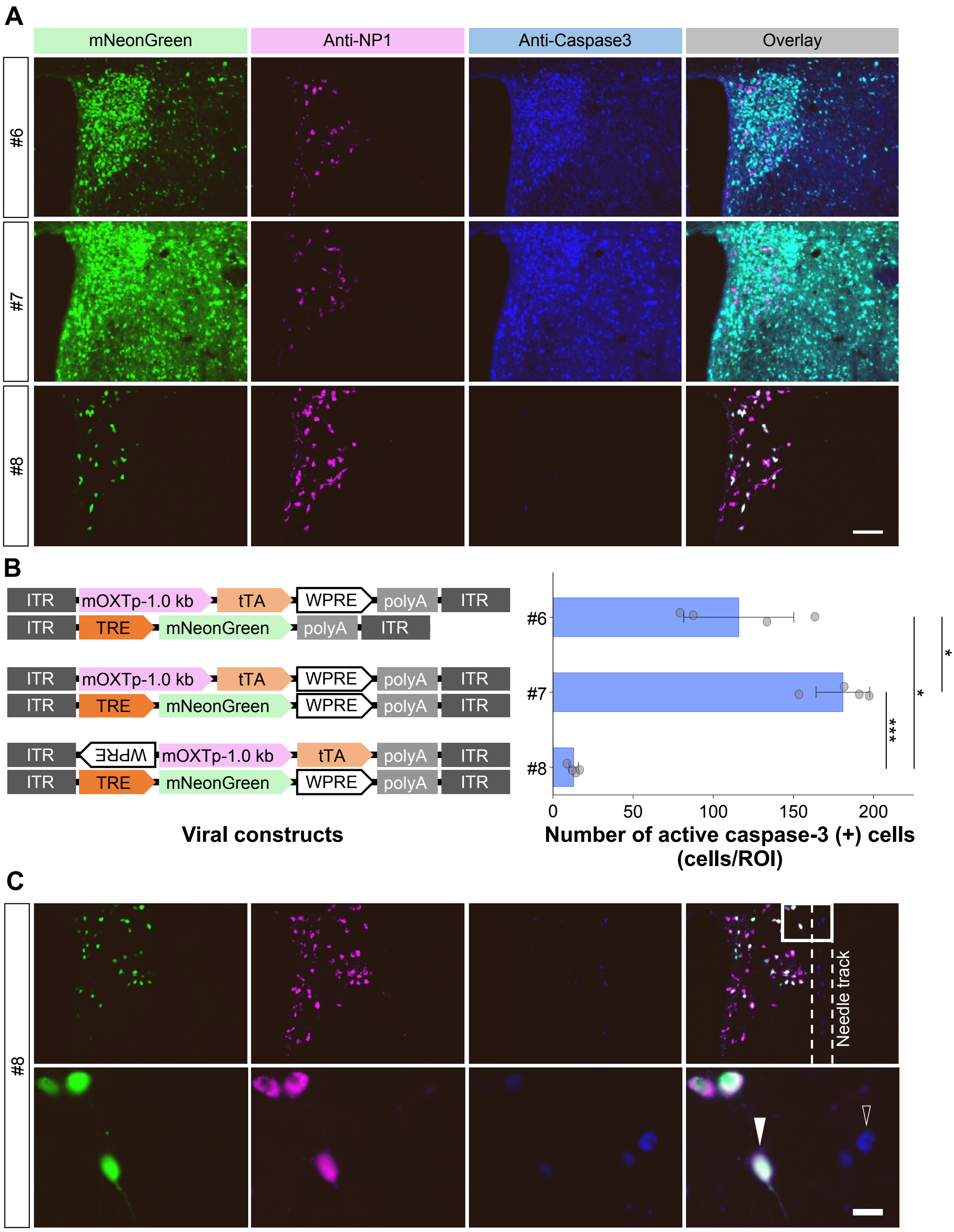

Figure S2. Toxic effect of excessive gene expression by tTA-WPRE-mediated amplification (related to Figure 1)

(A) Representative images showing the signals of mNG (green), NP1 immunofluorescence (magenta), and active caspase-3 immunofluorescence (blue). (B) Viral constructs and the number of active caspase-3-positive cells per FOV. (C) Representative images showing active caspase-3-positive cells in mice infected with the viral construct #8 (TAREGET-OXT). Active caspase-3-positive cells were restricted to the area adjacent to the syringe needle track. *p < 0.05; p<0.001***. Data are represented as mean ± SEM.

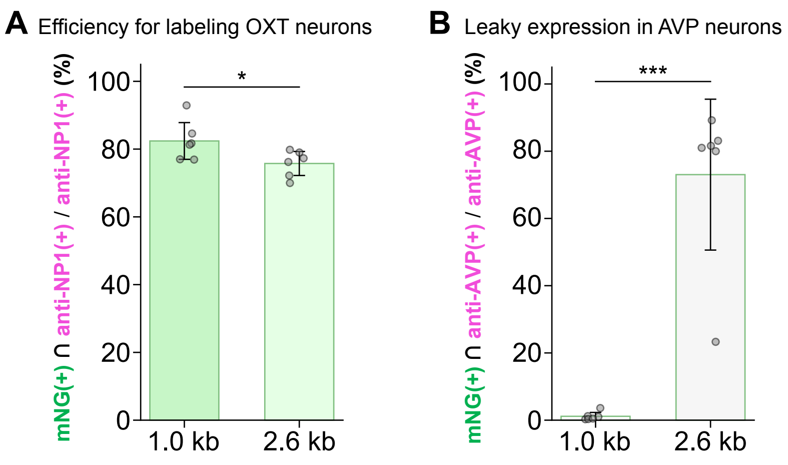

Figure S3. Efficiency of gene expression in OXT neurons and leaky expression in AVP neurons by the 1.0 kb and 2.6 kb mOXT promoters (related to Figure 2)

(A) Quantitative analysis of efficiency of gene expression in the OXT neurons. Percentage of the number of mNG and NP1 double-positive cells to that of NP1-positive cells was quantified. (B) Quantitative analysis of leaky gene expression in the AVP neurons. Percentage of the number of mNG and AVP double-positive cells to that of AVP-positive cells was quantified. The value of the 1.0 kb OXT promoter for labeling efficiency in OXT neurons in Figure 1 and that for leaky expression in AVP neurons in Figure 2 were reused for comparison in A and B, respectively. $^{*}p < 0.05$; $^{***}p < 0.001$. Data are represented as mean ± SEM. Scale bar, 200 μm (A) and 100 μm (D).

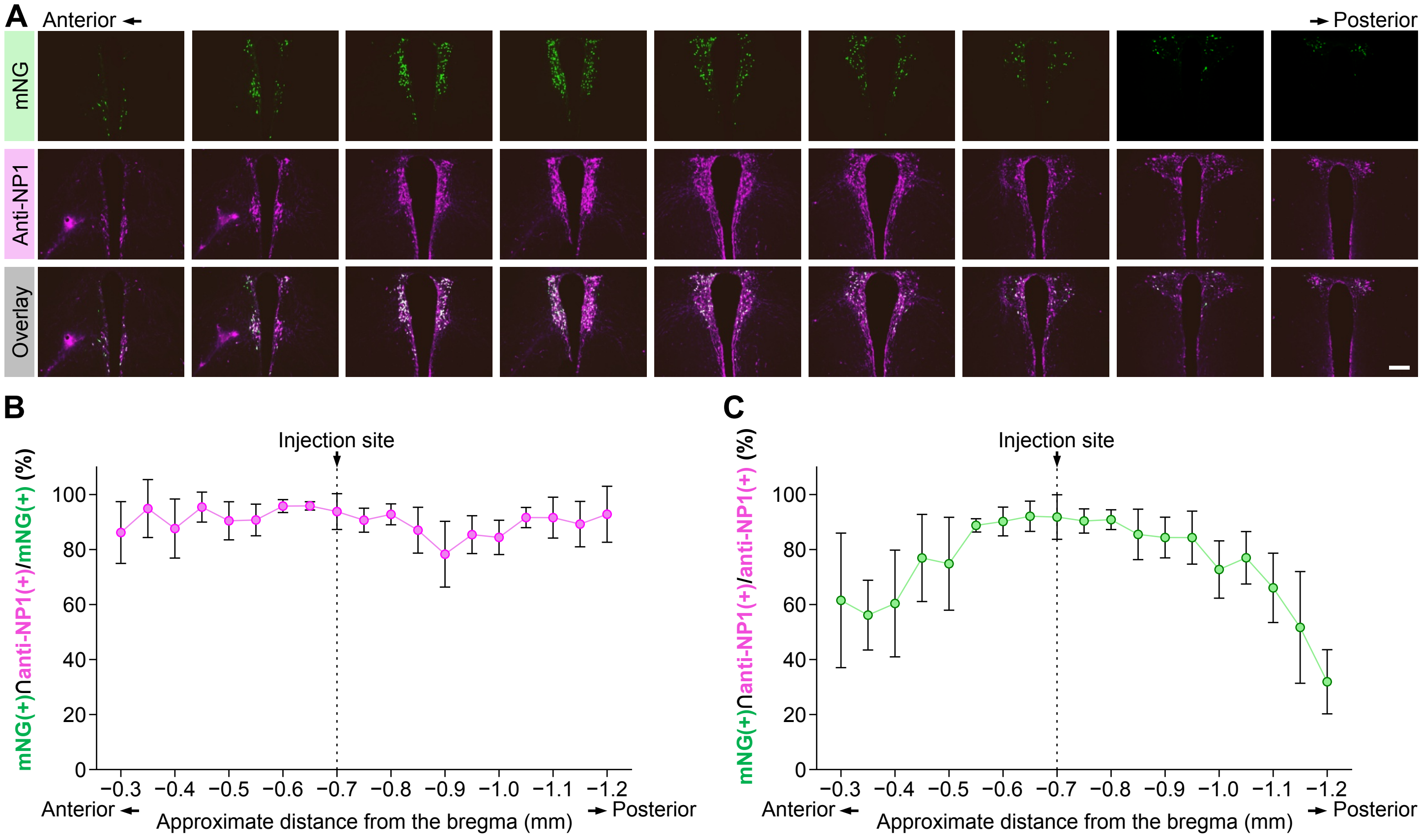

Figure S4. AAV TAREGET-OXT shows selective and efficient labeling along the anteroposterior axis of the PVH (related to Figure 2)

(A) Representative serial section images of AAV TAREGET-OXT-mediated mNG expression along the anteroposterior axis. The 25-μm-thick coronal sections were presented at 75 μm intervals from posterior (top left) to anterior (bottom right). Non-specific expressions in unintended cells were rarely observed. (B) Quantitative analysis of the specificity of mNG expression in OXT neurons across anteroposterior axis. Percentage of the number of mNG and NP1 double-positive cells to that of mNG-positive cells was quantified. (C) Quantitative analysis of the efficiency of mNG expression in OXT neurons across anteroposterior axis. Percentage of the number of mNG and NP1 double-positive cells to that of NP1-positive cells was quantified. Data are represented as mean ± SEM. Scale bar, 200 μm.

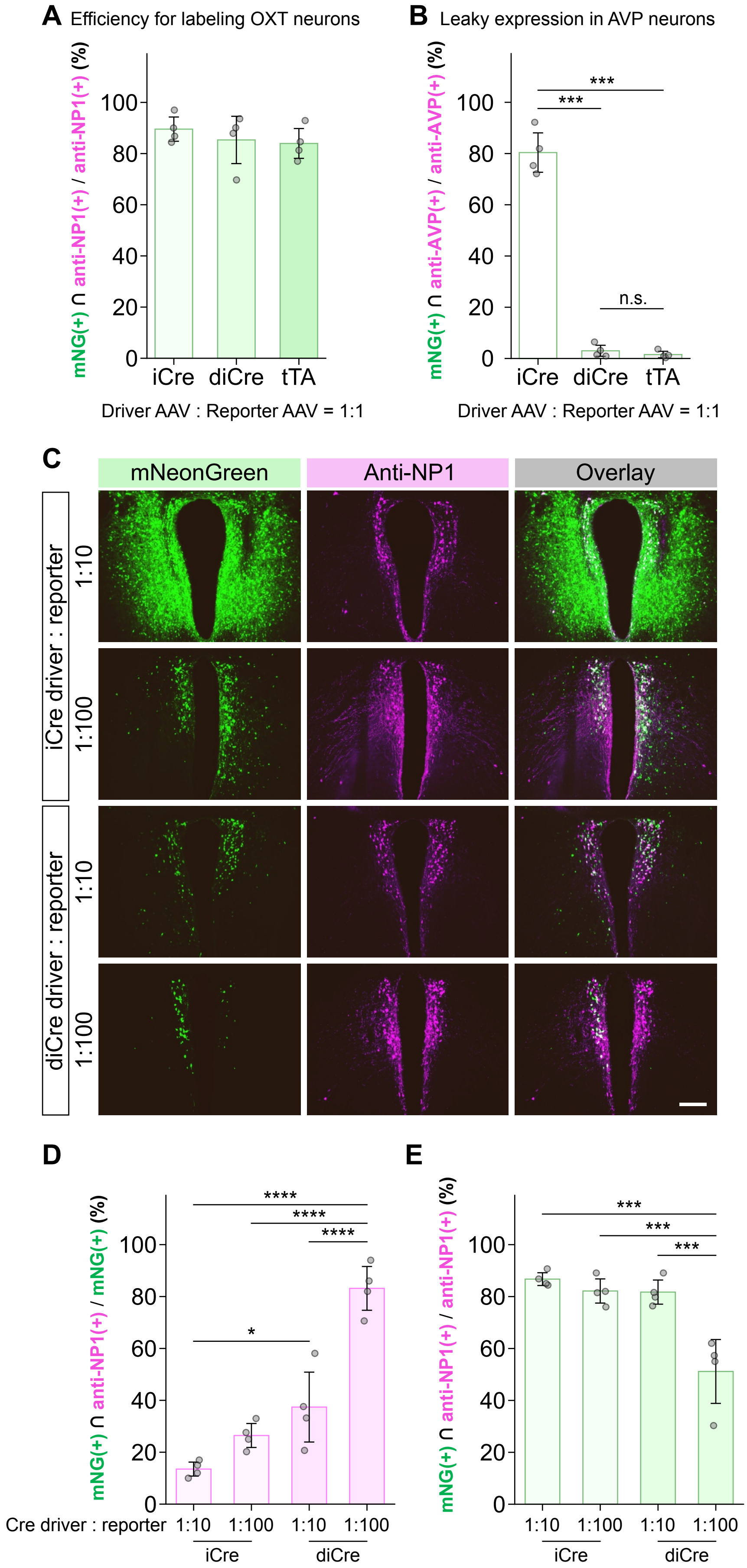

Figure S5. (legend on next page)

Figure S5. Optimization of viral titer improves cell-type specificity of Cre-mediate expression but reduces efficiency of labeling (related to Figure 3)

(A) Quantitative analysis of efficiency of gene expression in the OXT neurons. Percentage of the number of mNG and NP1 double-positive cells to that of NP1-positive cells was quantified. (B) Quantitative analysis of leaky gene expression in the AVP neurons. Percentage of the number of mNG and AVP double-positive cells to that of AVP-positive cells was quantified. The value of the tTA driver AAV for labeling efficiency in OXT neurons in Figure 1 and that for leaky expression in AVP neurons in Figure 2 were reused for comparison in A and B respectively. (C) Representative images of Cre-mediated mNG expression in the PVH with different viral titers. The Cre-driver AAVs were injected at the ratio of 1:10 and 1:100 relative to the reporter AAV (Cre-driver AAV vs. reporter AAV). All images were acquired at the same exposure time. Scale bar, 200 μm. (D) Quantification of specificity of mNG expression in the OXT neurons at the different ratios of the Cre-driver AAV vs. reporter AAV. (E) Quantification of efficiency of mNG expression in the OXT neurons. Optimization of the viral titer ratio for the diCre system resulted in higher specificity to the OXT neurons but reduced efficiency (sparser labeling). Scale bar, 200 μm. *$p < 0.05$; ***$p < 0.001$; ****$p < 0.0001$; n.s., not significant. Data are represented as mean ± SEM.

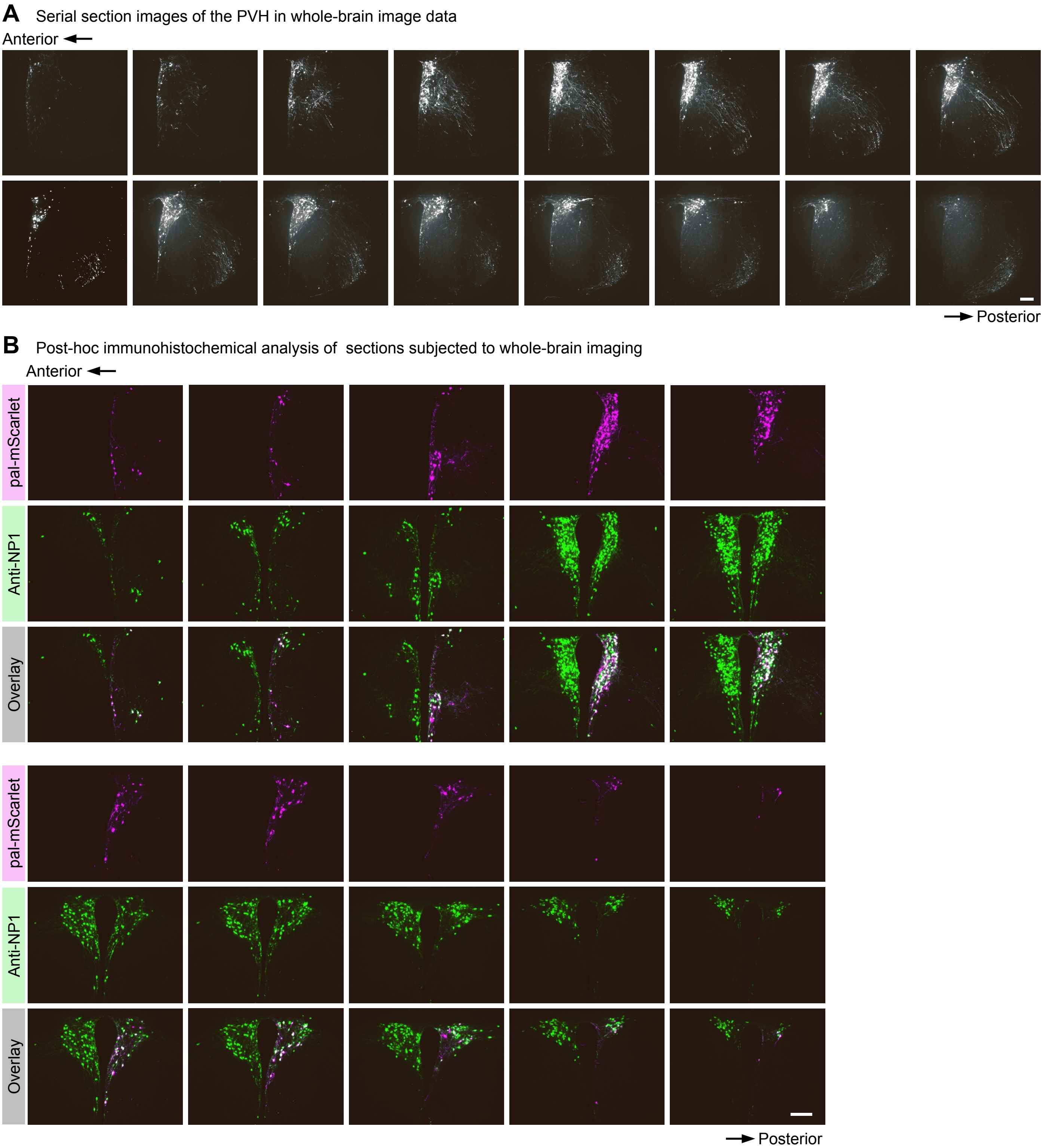

Figure S6. OXT neurons were selectively labeled with pal-mScarlet in the brain (related to Figure 4)

(A) Magnification of the PVH-adjacent regions in whole-brain imaging data. (B) Representative serial section images of post-hoc immunohistochemical verification. The result of a different mouse from A was shown here. The pal-Scarlet expression was restricted to the OXT neurons throughout the anteroposterior axis. Scale bar, 200 µm.

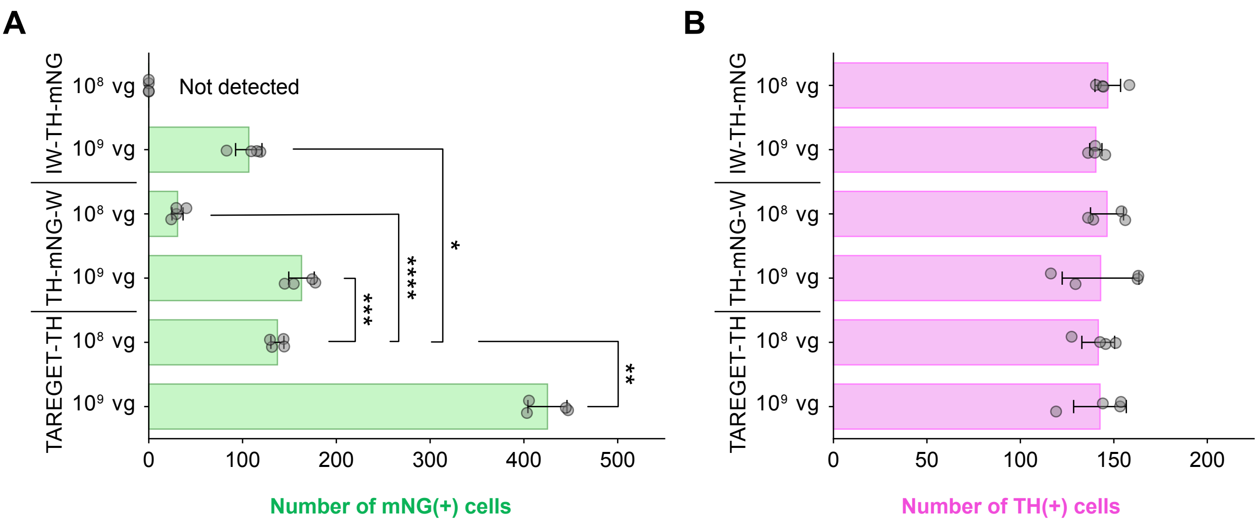

Figure S7. The number of mNeonGreen-positive cells by stereotaxic injection of TAREGET-TH (related to Figure 5)

(A) The number of mNG-positive neurons in the VTA. (B) The number of TH-positive neurons in the analyzed area. Cell numbers were calculated as a mean of cells per FOV in the single coronal section. $*p < 0.05$; $**p < 0.01$; $p<0.0001****$. Statistical significance is shown only for the comparisons between the optimized TAREGET-TH (10^8 vg) and other groups to avoid redundancy. Full statistics are shown in Table S1. Data are represented as mean ± SEM.

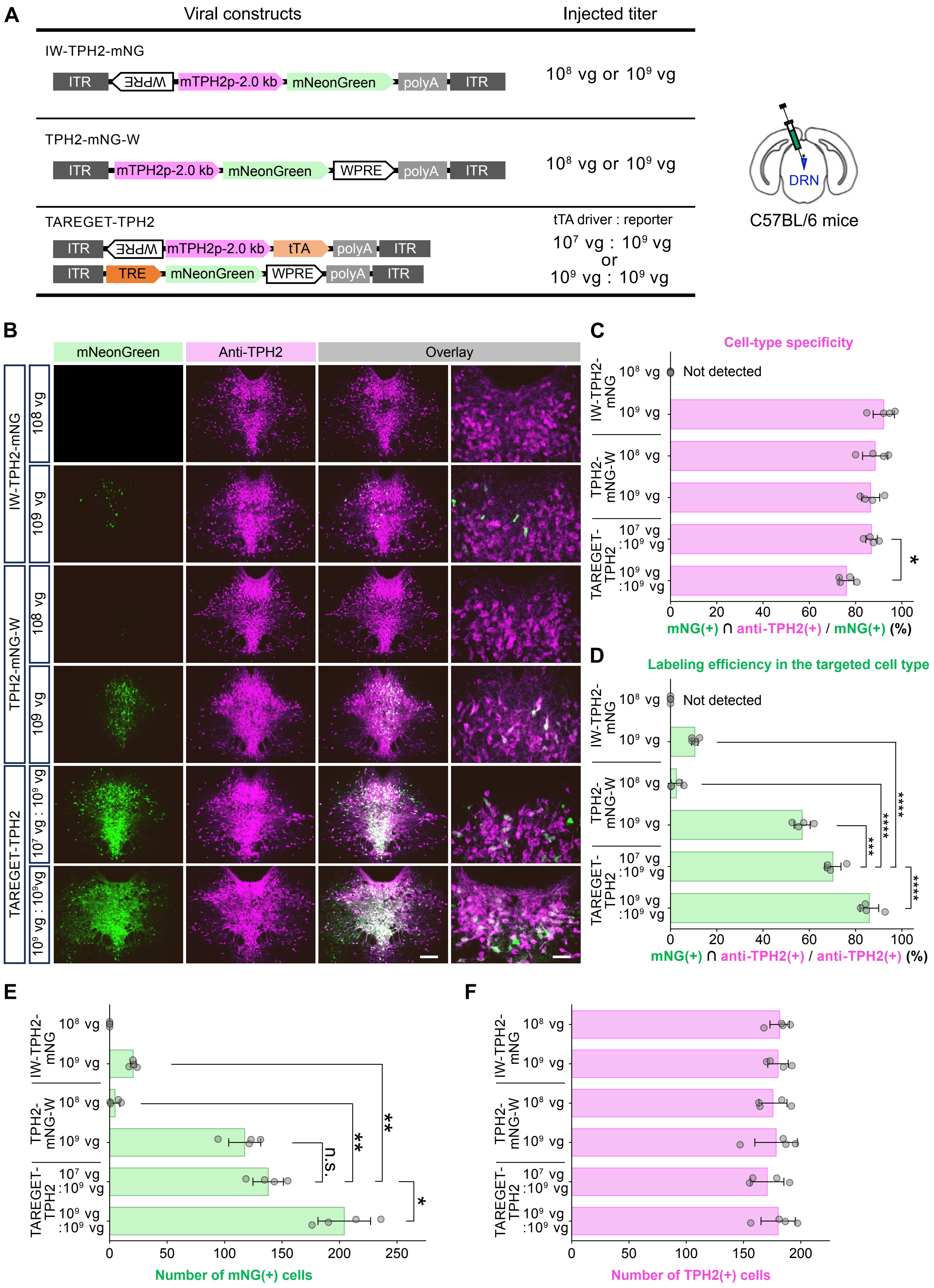

Figure S8. (legend on next page)

Figure S8. Application of the TAREGET strategy for labeling serotonergic neurons in the dorsal raphe nuclei (related to Figure 5)

(A) Scheme of the AAV vector constructs for selective gene expression in serotonergic neurons in the DRN using mTPH2 promoter. (B) Representative images of the DRN immunostained for TPH2 (serotonergic neurons). Each AAV group was injected into the DRN at two different viral doses indicated in (A). (C) Quantification of the specificity of mNG expression to serotonergic neurons. Percentage of mNG and TPH2 double-positive cells among mNG-positive cells was quantified. (D) Quantification of the efficiency of mNG expression in serotonergic neurons. Percentage of mNG and TPH2 double-positive cells among TPH2-positive cells was quantified. (E) The number of mNG-positive neurons in the DRN. (F) The number of TPH2-positive neurons in the analyzed area. DRN, dorsal raphe nuclei. Scale bar, 200 μm (B, left images in the overlays) and 50 μm (B, the right image in the overlay). The cell numbers were calculated as a mean of cells per FOV in the single coronal section. *$p < 0.05$; **$p < 0.01$; ***$p < 0.001$; $p<0.0001$****. Statistical significance is shown only for the comparisons between the optimized TAREGET-TPH2 (10^7 vg : 10^9 vg) and other groups to avoid redundancy. Full statistics are shown in Table S1. Data are represented as mean ± SEM.

Table S1. Results of statistical analysis

Figures

Figure 1C
Cell-type specificity
One-way ANOVA F(4,25) = 439.1 p = 9.21E-23
P value in multiple comparison

	#2	#5	#6	#7	#8
#2					
#5	0.00011				
#6	< 2e-16	< 2e-16			
#7	< 2e-16	< 2e-16	0.08331		
#8	0.81999	0.00015	< 2e-16	< 2e-16	

Figure 1D
Labeling efficiency
One-way ANOVA F(4,25) = 34.64 p = 7.37E-10
P value in multiple comparison

	#2	#5	#6	#7	#8
#2					
#5	0.33217				
#6	0.02405	0.00154			
#7	0.00409	0.00007	0.33814		
#8	0.04370	0.04673	0.00081	6.28E-05	

Figure 2C
Cell-type specificity
P value by Welch's t-test p = 9.76E-05

Figure 2D
Leakage in AVP neurons
P value by Welch's t-test p = 0.00075

Figure 3C
Cell-type specificity
One-way ANOVA F(2,9) = 343.5 p = 3.18E-09
P value in multiple comparison

	iCre	diCre	tTA
iCre			
diCre	0.00024		
tTA	3.70E-09	2.62E-08	

Figure 3E
Leaky expression in AVP neurons
One-way ANOVA F(2,9) = 35.28 p = 5.51E-05
P value in multiple comparison

	iCre	diCre	tTA
iCre			
diCre	0.00012		
tTA	0.00012	0.79769	

Figure 4F
Cell-type specificity
P value by Welch's t-test p = 0.32857

Figure 4G
Labeling efficiency
P value by Welch's t-test p = 0.20776

Figure 5C
Cell-type specificity
One-way ANOVA F(4,15) = 142.3 p = 9.77E-12
P value in multiple comparison

	IW-TH-mNG 10^9	TH-mNG-W 10^8	TH-mNG-W 10^9	TAREGET-TH 10^8	TAREGET-TH 10^9
IW-TH-mNG 10^9					
TH-mNG-W 10^8	3.40.E-08				
TH-mNG-W 10^9	3.60.E-05	0.00025			
TAREGET-TH 10^8	4.40.E-08	0.73102	0.00032		
TAREGET-TH 10^9	4.40.E-06	3.40.E-11	2.40.E-09	4.00.E-11	

Figure 5D
Labeling efficiency
One-way ANOVA F(4,15) = 413 p = 3.82E-15
P value in multiple comparison

	IW-TH-mNG 10^9	TH-mNG-W 10^8	TH-mNG-W 10^9	TAREGET-TH 10^8	TAREGET-TH 10^9
IW-TH-mNG 10^9					
TH-mNG-W 10^8	2.50.E-08				
TH-mNG-W 10^9	4.00.E-11	5.40.E-14			
TAREGET-TH 10^8	2.80.E-11	4.40.E-14	1.00000		
TAREGET-TH 10^9	2.80.E-11	4.40.E-14	1.00000	1.00000	

Figure 6C
Cell-type specificity
P value by Student's t-test p = 0.00095

Figure 6D
Labeling efficiency
P value by Student's t-test p = 0.40591

Figure 6E
NeuN knockdown
One-way ANOVA F(2,9)=276 p = 8.38E-09
P value in multiple comparison

	tTA only	CMV-saCas9	TAREGET-TH::saCas9
tTA only			
CMV-saCas9	4.62E-07		
TAREGET-TH::saCas9	6.90E-09	5.20E-06	

Supplementary Figures

Figure S1B

Number of mNG(+) neurons

One-way ANOVA $F(4,25) = 34.9$ $p = 6.82E-10$

P value in multiple comparison

	#2	#5	#6	#7	#8
#2					
#5	0.19283				
#6	0.03205	0.03903			
#7	0.00375	0.00501	0.04053		
#8	0.13025	0.97150	0.04677	4.46E-03	

Figure S1C

Number of NP1(+) neurons

One-way ANOVA $F(7,40) = 7.529$ $p = 8.86E-06$

P value in multiple comparison

	#1	#2	#3	#4	#5	#6	#7	#8
#1								
#2	1.00000							
#3	1.00000	1.00000						
#4	1.00000	1.00000	1.00000					
#5	1.00000	1.00000	1.00000	1.00000				
#6	0.00929	0.00668	0.00900	0.00950	0.00409			
#7	0.00157	0.00108	0.00150	0.00164	0.00061	1.00000		
#8	1.00000	1.00000	1.00000	1.00000	1.00000	0.02478	0.00441	

Figure S2B

Number of active caspase-3(+) cells

One-way ANOVA $F(2,9) = 44.01$ $p = 2.26E-05$

P value in multiple comparison

	#6	#7	#8
#6			
#7	0.03793		
#8	0.02695	0.00092	

Figure S3A

Labeling efficiency in OXT neurons

P value by Welch's t-test $p = 0.03502$

Figure S3B

Leaky expression in AVP neurons

P value by Welch's t-test $p = 0.00081$

Figure S5A

Labeling efficiency in OXT neurons

One-way ANOVA $F(2,9)=0.776$ $p = 0.48900$

P value in multiple comparison

	iCre	diCre	tTA
iCre			
diCre	0.89000		
tTA	0.75000	0.89000	

Figure S5B

Leaky expression in AVP neurons

One-way ANOVA $F(2,9) = 19.35$ $p = 0.00055$

P value in multiple comparison

	iCre	diCre	tTA
iCre			
diCre	0.00059		
tTA	0.00059	0.13495	

Figure S5D

Cell-type specificity

One-way ANOVA $F(3,12) = 39.38$ $p = 1.73E-06$

P value in multiple comparison

	iCre 1:10	iCre 1:100	diCre 1:10	diCre 1:100
iCre 1:10				
iCre 1:100	0.162			
diCre 1:10	0.013	0.162		
diCre 1:100	1.70E-06	1.29E-05	8.87E-05	

Figure S5D

Labeling efficiency

One-way ANOVA $F(3,12) = 16.34$ $p = 0.00015$

P value in multiple comparison

	iCre 1:10	iCre 1:100	diCre 1:10	diCre 1:100
iCre 1:10				
iCre 1:100	1			
diCre 1:10	1	1		
diCre 1:100	0.00029	0.00063	0.00075	

Figure S7A

Number of mNG(+) cells

One-way ANOVA $F(4,15) = 373$ $p = 8.13E-15$

P value in multiple comparison

	IW-TH-mNG 10^9	TH-mNG-W 10^8	TH-mNG-W 10^9	TAREGET-TH 10^8	TAREGET-TH 10^9
IW-TH-mNG 10^9					
TH-mNG-W 10^8	0.00002				
TH-mNG-W 10^9	4.40E-06	1.40E-09			
TAREGET-TH 10^8	0.01390	3.70E-07	0.00028		
TAREGET-TH 10^9	1.20E-13	5.70E-15	9.50E-12	4.70E-13	

Figure S7B
Number of TH(+) cells
One-way ANOVA F(5,18) = 0.723 p = 0.615
P value in multiple comparison

	IW-TH-mNG 10^8	IW-TH-mNG 10^9	TH-mNG-W 10^8	TH-mNG-W 10^9	TAREGET-TH 10^8	TAREGET-TH 10^9
IW-TH-mNG 10^8						
IW-TH-mNG 10^9	1.00000					
TH-mNG-W 10^8	1.00000	1.00000				
TH-mNG-W 10^9	1.00000	1.00000	1.00000			
TAREGET-TH 10^8	1.00000	1.00000	1.00000	1.00000		
TAREGET-TH 10^9	1.00000	1.00000	1.00000	1.00000	1.00000	

Figure S8C
Cell-type specificity
One-way ANOVA F(4,15) = 6.523 p = 3.01E-03
P value in multiple comparison

	IW-TPH2-mNG 10^9	TPH2-mNG-W 10^8	TPH2-mNG-W 10^9	TAREGET-TPH2 10^7	TAREGET-TPH2 10^9
IW-TPH2-mNG 10^9					
TPH2-mNG-W 10^8	1.00000				
TPH2-mNG-W 10^9	0.64660	1.00000			
TAREGET-TPH2 10^7	0.66140	1.00000	1.00000		
TAREGET-TPH2 10^9	0.00210	0.01800	0.04720	0.04230	

Figure S8D
Labeling efficiency
One-way ANOVA F(4,15) = 423.4 p = 3.18E-15
P value in multiple comparison

	IW-TPH2-mNG 10^9	TPH2-mNG-W 10^8	TPH2-mNG-W 10^9	TAREGET-TPH2 10^7	TAREGET-TPH2 10^9
IW-TPH2-mNG 10^9					
TPH2-mNG-W 10^8	0.00676				
TPH2-mNG-W 10^9	5.80E-11	7.00E-12			
TAREGET-TPH2 10^7	2.10E-12	3.70E-13	0.00019		
TAREGET-TPH2 10^9	8.50E-14	2.10E-14	3.20E-08	5.00E-05	

Figure S8E
Number of mNG(+) cells
One-way ANOVA F(4,15) = 114.2 p = 4.83E-11
P value in multiple comparison

	IW-TPH2-mNG 10^9	TPH2-mNG-W 10^8	TPH2-mNG-W 10^9	TAREGET-TPH2 10^7	TAREGET-TPH2 10^9
IW-TPH2-mNG 10^9					
TPH2-mNG-W 10^8	0.00821				
TPH2-mNG-W 10^9	0.00378	0.00289			
TAREGET-TPH2 10^7	0.00303	0.00157	0.11511		
TAREGET-TPH2 10^9	0.00363	0.00278	0.02328	0.01631	

Figure S7B
Number of TPH2(+) cells
One-way ANOVA F(5,18) = 0.269 p = 0.942
P value in multiple comparison

	IW-TPH2-mNG 10^8	IW-TPH2-mNG 10^9	TPH2-mNG-W 10^8	TPH2-mNG-W 10^9	TAREGET-TPH2 10^7	TAREGET-TPH2 10^9
IW-TPH2-mNG 10^8						
IW-TPH2-mNG 10^9	1.00000					
TPH2-mNG-W 10^8	1.00000	1.00000				
TPH2-mNG-W 10^9	1.00000	1.00000	1.00000			
TAREGET-TPH2 10^7	1.00000	1.00000	1.00000	1.00000		
TAREGET-TPH2 10^9	1.00000	1.00000	1.00000	1.00000	1.00000	

Table S1. Result of statistical analysis (related to STAR Methods)

The detailed results of statistical analysis were indicated.
